# A soft, ultra-tough and multifunctional artificial muscle for volumetric muscle loss treatment

**DOI:** 10.1093/nsr/nwae422

**Published:** 2024-11-22

**Authors:** Peng-Fei Qiu, Lei Qiang, Weiqing Kong, Fang-Zhou Wang, Hong-Qin Wang, Ke-Xin Hou, Yihao Liu, Cheng-Hui Li, Pengfei Zheng

**Affiliations:** State Key Laboratory of Coordination Chemistry, School of Chemistry and Chemical Engineering, Collaborative Innovation Center of Advanced Microstructures, Nanjing University, Nanjing 210023, China; Department of Orthopaedic Surgery, Children's Hospital of Nanjing Medical University, Nanjing 210004, China; Department of Orthopedic Surgery, Xuzhou Central Hospital, Xuzhou Clinical School of Xuzhou Medical University, Xuzhou 221009, China; State Key Laboratory of Coordination Chemistry, School of Chemistry and Chemical Engineering, Collaborative Innovation Center of Advanced Microstructures, Nanjing University, Nanjing 210023, China; State Key Laboratory of Coordination Chemistry, School of Chemistry and Chemical Engineering, Collaborative Innovation Center of Advanced Microstructures, Nanjing University, Nanjing 210023, China; State Key Laboratory of Coordination Chemistry, School of Chemistry and Chemical Engineering, Collaborative Innovation Center of Advanced Microstructures, Nanjing University, Nanjing 210023, China; Shanghai Key Laboratory of Orthopedic Implant, Department of Orthopedic Surgery, Shanghai Ninth People's Hospital, Shanghai Jiao Tong University School of Medicine, Shanghai 200011, China; State Key Laboratory of Coordination Chemistry, School of Chemistry and Chemical Engineering, Collaborative Innovation Center of Advanced Microstructures, Nanjing University, Nanjing 210023, China; Department of Orthopaedic Surgery, Children's Hospital of Nanjing Medical University, Nanjing 210004, China

**Keywords:** volumetric muscle loss, scaffolds, shape-memory polymer, skeletal muscle, stimuli responsiveness

## Abstract

The escalating prevalence of skeletal muscle disorders highlights the critical need for innovative treatments for severe injuries such as volumetric muscle loss. Traditional treatments, such as autologous transplants, are constrained by limited availability and current scaffolds often fail to meet complex clinical needs. This study introduces a new approach to volumetric muscle loss treatment by using a shape-memory polymer (SMP) based on block copolymers of perfluoropolyether and polycaprolactone diol. This SMP mimics the biomechanical properties of natural muscle, exhibiting a low elastic modulus (2–6 MPa), high tensile strength (72.67 ± 3.19 MPa), exceptional toughness (742.02 ± 23.98 MJ m^−3^) and superior biocompatibility, thereby enhancing skeletal muscle tissue integration and regeneration within 4 weeks. Moreover, the polymer's shape-memory behavior and ability to lift >5000 times its weight showcase significant potential in both severe muscle disorder treatment and prosthetic applications, surpassing existing scaffold technologies. This advancement marks a pivotal step in the development of artificial muscles for clinical use.

## Teaser

A novel shape memory polymer mimics the properties of natural muscles, offering new hope for the treatment of severe muscle injuries and the enhancement of prosthetics with its remarkable strength, toughness, and biocompatibility.

## INTRODUCTION

**Figure 1.**
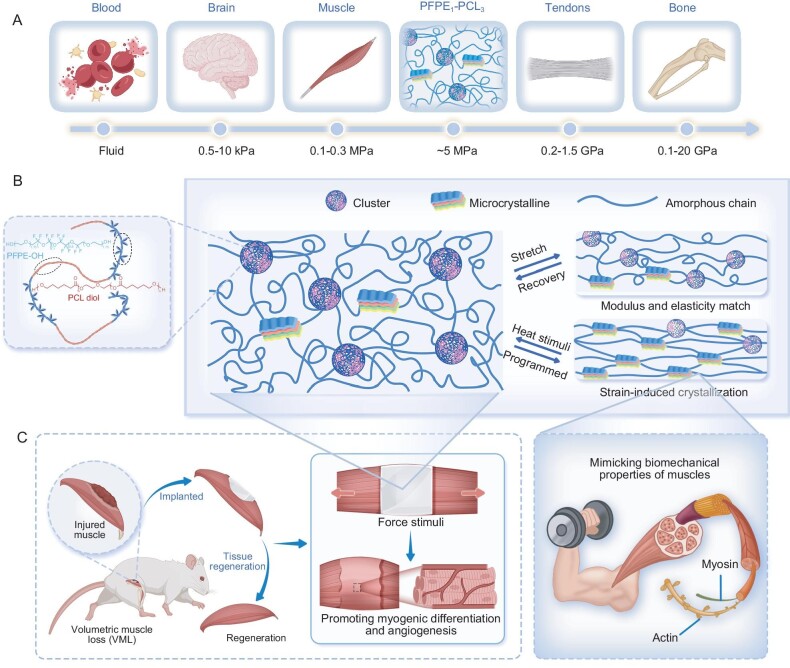
Multifunctional artificial muscles with tissue-like modulus achieved through biomimetic design. (A) Summary plot of the range of elastic moduli for PFPE_1_–PCL_3_ and representative biological tissues. (B) Schematic structure of the designed multifunctional artificial muscle. (C) Multifunctional artificial muscles can treat volumetric muscle loss, promote myogenic differentiation and angiogenesis (left) and mimic natural muscle function (right).

Volumetric muscle loss (VML) is a massive loss of skeletal muscle due to traumatic events, surgical ablation, congenital defects, tumor removal or denervation [1]. As the volume of muscle loss exceeds the self-regenerate ability of skeletal muscle, VML causes severe functional disability or impairments and thus reduce patients’ quality of life [1]. Currently, autologous muscle tissue transplantation is the gold standard for treating VML in clinics. However, due to the limited muscle volume of the donor site and low muscle regeneration efficiency after transplantation, the functional recovery of the VML muscle remains far from being satisfactory [2–4]. Recent advances in tissue engineering have led researchers to develop biomimetic scaffolds for skeletal muscle regeneration [1,5,6] or prostheses for amputees [7,8]. Key among these are innovations in soft materials with mechanical adaptability to biological tissues (Fig. 1A), such as biomaterial scaffolds [9,10] and artificial muscles [11–13], which are designed to exhibit high biocompatibility, robust mechanical properties, enhanced tissue regenerative capabilities and the ability to imitate limb movement.

So far, various scaffold materials, including natural polymers such as collagen and alginate [9,14,15] and synthetic polymers such as hydrogels [16–18] and elastomers [19–21], have been developed. However, most of them failed to meet complex clinical demands for biological activity. Moreover, they typically lack the necessary dynamic responsiveness to mimic the complex movements and force outputs that are characteristic of natural muscles, and thus are limited in application as prostheses for amputees. An ideal scaffold material for VML treatment should possess mechanical and biochemical properties that are akin to those of muscle tissue (Fig. 1A), including biocompatibility, appropriate elasticity, toughness and mechanical strength to support normal muscle activities at the early stage (muscle contraction and strength recovery). It should aid in daily function recovery to stimulate muscle growth (Fig. 1C), preventing muscle atrophy and joint dysfunction caused by traditional surgical suturing and plaster fixation, especially for large mammals in clinical applications. To direct skeletal muscle growth, the biomimetic scaffolds should also facilitate cell alignment, promote skeletal muscle formation and stimulate vascularization and innervation (Fig. 1C). Moreover, to achieve precise motion control and functional recovery, artificial muscles must optimally balance strain, stress, energy density and mechanical strength to mimic sophisticated biological movements and power prosthetics for amputees [11,12]. Therefore, developing a multifunctional material capable of both promoting VML skeletal muscle regeneration and acting as a prosthetic limb actuator holds significant clinical potential.

However, achieving these capabilities often involves a combination of mutually exclusive properties into a single material, leading to huge challenges in material design. To ensure successful implantation and facilitate the effective attachment and growth of muscle cells, an elastic modulus that is comparable to that of natural muscle is required to guarantee both mechanical compatibility and excellent biocompatibility [22–25]. On the other hand, however, in order to provide high actuation force and energy/power density and improve the resilience toward mechanical damage, the actuator materials need to be sufficiently tough. The dual requirements of mechanical robustness and low elastic modulus often conflict, as soft materials always suffer from poor mechanical strength due to the low cross-linking density or low cross-linking bond energy [26,27]. Moreover, achieving dynamic responsiveness for precise motion control requires the incorporation of stimuli-responsive units [8,25,28]. However, the presence of stimuli-responsive units (such as shape-memory and liquid-crystal elastomers) will cause unsatisfactory environmental stability and cycling performance [24,29–32]. In cases of bioactivity, chemical modification is always necessary, as most natural polymers do not have sufficient properties. However, chemical modification generally introduces toxic moieties, which is unfavorable for biocompatibility. Addressing these contradictions requires innovative material design and engineering to balance the dual demands of formability and functional performance in artificial muscles.

Biological materials exhibit great superiority in tailoring the extreme mechanical properties to simultaneously fulfill different functions. As an example, muscle tissues combine the properties of high mechanical strength, high stretchability, excellent tear resistance and resilience while still being soft [33–36]. The mechanism behind such amazing phenomena is their ability to modulate the hierarchical structures and molecular interactions. In this study, by taking inspiration from the structure and function of muscles, we have successfully developed a multifunctional artificial muscle by using a shape-memory polymer (SMP) made from block copolymers of perfluoropolyether (PFPE–OH) and polycaprolactone (PCL) diol (Fig. 1B). Our synthesized polymer mirrors natural muscle tissue with a low elastic modulus (2–6 MPa), ultra-high tensile strength (40–70 MPa), exceptional strain-at-break (2100%–2300%) and ultra-high toughness (450–750 MJ m^−3^). Moreover, the optimal elastomer showcases superior actuation performance and mechanical-training-reinforcement characteristics that similar to those of natural muscles (Fig. 1C). An actuator made from this SMP, with ultra-high energy density, can lift an object that is >5000 times its own weight with reversible actuation. This new artificial muscle can be implanted *in vivo* as a scaffold material for treating VML diseases and can also function as artificial muscle to drive prosthetics. The multifunctionality and high efficiency of this artificial muscle highlight its importance in regenerative medicine and biomedical materials.

## RESULTS AND DISCUSSION

### Design, synthesis and general characterizations

The muscle fibers in skeletal muscles are highly organized in a parallel arrangement, which contributes to their efficient force transmission. The connective tissues surrounding these muscle fibers, which are rich in collagen, exhibit a disordered and wavy morphology, ensuring superior softness and flexibility. Under stress and deformation, they realign and reconstruct intermolecular interactions, providing remarkable tear resistance and mechanical strength [35,37]. By adjusting the spatial location through the relative slide between actin and myosin, the skeletal muscles can achieve reversible actuation and enable us to move or lift heavy objects. More importantly, skeletal muscles can remodel and strengthen themselves by mechanical training to satisfy the mechanical requirement due to the destruction and reconstruction of the muscle fibrils. These phenomena indicate that the formation of hierarchical structures is an effective method for enhancing the mechanical properties of polymer materials and providing specific functions [38–41]. By precisely adjusting the interactions between the polymer segments, it is possible to achieve excellent comprehensive properties that are comparable to those of natural materials, including high mechanical strength, softness and multifunctionality. As block polymers have been shown to exhibit interesting self-assembly and microphase-separation structures, while the formation of self-assembled and microphase-separated structures has been proven to be an effective method for enhancing the mechanical properties of polymer materials [42], here we choose block polymers for our design.

PCL is widely recognized as a biocompatible SMP with numerous commercial applications. However, its high crystallinity results in substantial stiffness [43], making PCL less suitable for use in body-compliant devices or tissue engineering, where high adaptability and conformability are crucial. Additionally, the inherent one-way shape-memory property of PCL limits its utility in the development of artificial muscles. PFPE was selected as the soft block as it has very low *T*_g_ (below −100°C) and has excellent tribological properties due to the intrinsically small intermolecular friction [44]. It also demonstrates significant potential and advantages in the biomedical field due to excellent biocompatibility, mechanical compatibility, chemical stability, stretchability, low modulus and long-term stability [45–49]. On the other hand, the CF_2_ has a large van der Waals volume [50], while the presence of polar C–F or CF_2_ units can bring inter/intramolecular dipole–dipole interactions within the neighboring dipoles (C–F, CF_2_, C–O–C, C=O, etc.), which facilitates the formation of disordered and amorphous structures (Fig. 1B and Fig. S1). We envisage that the relatively larger molecular volume, weakened PCL inter/intra-chain stacking and reduced inter-chain friction may enhance the reversible sliding between polymer chains, preventing PCL segments from forming stable and large crystals, thus improving elasticity. Moreover, due to the inter/intramolecular dipole–dipole interactions between PFPE and PCL, the resulting polymer may self-organize into a highly disordered state with self-assembled and microphase-separated structures. Isocyanate groups were used to react with the hydroxy group and prepare the block polymer, as polyurethane has been proven to be effective for constructing polymers with regularly interspaced soft and hard segments (Fig. S1).

**Figure 2.**
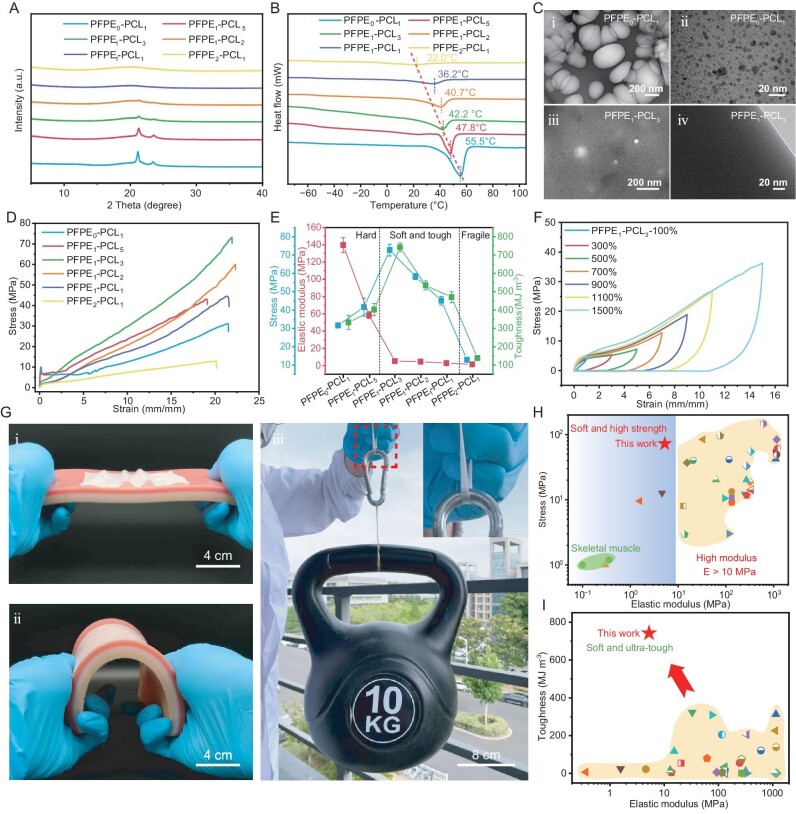
General characterization and mechanical properties of PFPE*_x_*–PCL*_y_*. (A) XRD analysis of elastomers with varying molar ratios of PFPE–OH and PCL–OH. (B) Analysis conducted using a differential scanning calorimeter (DSC). (C) Transmission electron microscopy (TEM) images of (i) and (ii) PFPE_0_–PCL_1_ and (iii) and (iv) PFPE_1_–PCL_3_. (D) Stress–strain curves of PFPE*_x_*–PCL*_y_* and (E) corresponding stress, elastic modulus and toughness. (F) Cyclic stress–strain curves of PFPE_1_–PCL_3_ at various strain levels. (G) (i) and (ii) Photograph comparing the elastic modulus of PFPE_1_–PCL_3_ with that of artificial skin tissue and (iii) photograph showing 0.2 g of PFPE_1_–PCL_3_ elastomer lifting a 10-kg weight. (H) Comparison of the elastic modulus and tensile strength of PFPE_1_–PCL_3_ with those of shape-memory polymers reported in other literature. (I) Comparison of the elastic modulus and toughness of PFPE_1_–PCL_3_ with those of shape-memory polymers reported in other literature.

We controlled the feed ratio of PFPE–OH, PCL–OH and hexamethylene diisocyanate (HDI) and obtained a series of elastomers, noted as PFPE*_x_*–PCL*_y_* (*x* and *y* denote the molar ratios of PFPE and PCL in the elastomers, respectively), via a two-step reaction procedure (Fig. S2). Gel permeation chromatography (Table S1) and Fourier-transform infrared (FTIR) spectroscopy (Fig. S3) confirmed the successful synthesis of the elastomers. The temperature-dependent FTIR spectra of PFPE*_x_*–PCL*_y_* reveal that, as the temperature rises, the strong dipole–dipole interactions between the PFPE and PCL chains will dissociate (Fig. S4). Thermogravimetric analysis (TGA) indicates a decomposition temperature of ∼300°C (Fig. S5) and confirms the absence of solvent residues. According to the differential scanning calorimeter (DSC) and X-ray diffraction (XRD) results (Fig. 2A and B, Fig. S6 and Table S2), the introduction of PFPE prohibits PCL from forming stable and massive crystals and replaces them with microcrystals (low-content PFPE) and clusters (Fig. 1B). The inhibition of crystallization was further proved in the transmission electron microscopy (TEM) images (Fig. 2C(i)–(iv) and Fig. S7). The energy dispersive spectrometer elements analysis (Fig. S8) showed the uniform distribution of the F element, which contributed to the inhibition of crystallization.

Scanning electron microscopy (SEM) imaging captured the morphological changes in the PFPE*_x_*–PCL*_y_* series elastomers at varying PFPE and PCL ratios (Fig. S9). PFPE_0_–PCL_1_ exhibited uniform and smooth morphologies, indicating a homogeneous network distribution. The incorporation of PFPE led to phase separation in PFPE_1_–PCL_5_, but the resulting structure was non-uniform. The absence or non-uniformity of the self-assembled structures causes PCL segments to aggregate into crystalline domains, resulting in a rigid, non-elastic material. With the increased proportion of PFPE, the phase separation and self-assembled structures in the material became more pronounced and uniform, as observed in PFPE_1_–PCL_3_ and PFPE_1_–PCL_2_. When the proportion of PFPE surpassed a certain threshold, the morphology changed rapidly (PFPE_1_–PCL_1_ and PFPE_2_–PCL_1_). However, due to the non-crystalline nature and high flexibility of the PFPE chains, the material exhibited softer properties. Small-angle X-ray scattering (SAXS) further confirmed the self-assembled structure by evaluating the domain size (*d* = 2π/*q*, where *q* is the SAXS peak position). PFPE_0_–PCL_1_ showed a strong peak at *q* = 0.034 Å^−1^, corresponding to large crystalline domains formed by PCL chain stacking (*d* = 18.5 nm) (Fig. S10). With the introduction of PFPE, the peak at *q* = 0.034 Å^−1^ disappeared and new peaks emerged at around *q* = 0.06 and 0.13 Å^−1^, indicating that PFPE effectively suppressed PCL crystallization and promoted the formation of a self-assembled structure.

### Mechanical and training reinforcement properties

As shown in the stress–strain curve in Fig. 2D and Fig. S11, the elastic modulus of PFPE*_x_*–PCL*_y_* decreases gradually with increasing PFPE content (Fig. 2E and Table S3). The elastic modulus is 139.56 ± 8.68 MPa for PFPE_0_–PCL_1_ and 5.27 ± 0.05 MPa for PFPE_1_–PCL_3_, and drops to 1.57 ± 0.13 MPa for PFPE_2_–PCL_1_. PFPE_1_–PCL_3_ exhibits the best mechanical property, with a tensile strength of 72.67 ± 3.19 MPa and toughness of 742.02 ± 23.98 MJ m^−3^ (Fig. 2E and Table S3), which meets the modulus-matching requirements for *in vivo* implantation. Below, we will focus solely on the characterization of PFPE_1_–PCL_3_. Cyclic stress–strain curves at various strains were analysed by utilizing a graded multi-sample loading approach (Fig. 2F and Fig. S12A). As the strain increased, a marked transition to plastic behavior was observed, characterized by a rapid expansion in the hysteresis loop, particularly at higher strain levels, demonstrating its effective energy dissipation and storage capacity. To further verify this behavior, additional experiments that employed progressively increasing strains were conducted (Fig. S12B). Results from these experiments demonstrated that, beyond a 200% strain threshold, the plastic behavior of PFPE_1_–PCL_3_ became increasingly prominent, with significant accumulations of residual strain observed during cyclic testing, indicating its capacity for shape fixation as an SMP. We compared the modulus of PFPE_1_–PCL_3_ with that of artificial skin tissue. As shown in Fig. 2G(i), PFPE_1_–PCL_3_ effectively forms skin-like wrinkles and adapts to the bending of the skin (Fig. 2G(ii)). In contrast, classic PCL SMP exhibits significant rigidity and fails to match the mechanical behavior of human tissue (Fig. S13). PFPE_1_–PCL_3_ not only shows softness that is comparable to that of human skin tissue but also demonstrates remarkable toughness, with a 0.2-g sample able to lift 50 000 times its own weight (Fig. 2G(iii)). Compared with currently reported SMPs, our PFPE_1_–PCL_3_ maintains a low modulus while exhibiting tensile strength and toughness that are far superior to those previously reported (Fig. 2H and I).

**Figure 3.**
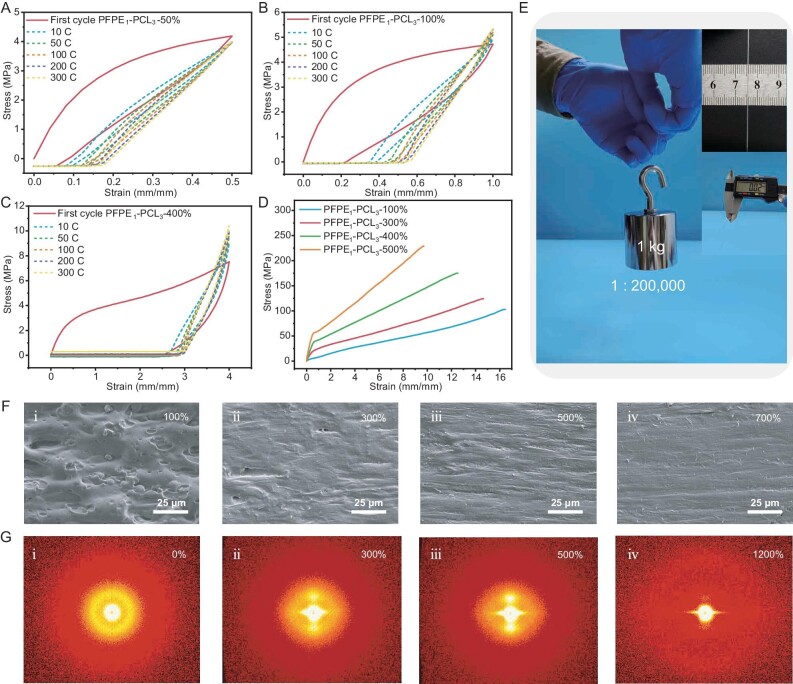
Training reinforcement properties of PFPE_1_–PCL_3_ and mechanism study. (A–C) Cyclic stress–strain curves of PFPE_1_–PCL_3_ under repetitive mechanical-training process at different strains showing self-strengthening properties with the increased cycles. (D) Stress–strain curves of the PFPE_1_–PCL_3_ after mechanical training at different strains for 300 cycles. (E) Strengthened fiber (weight 5 mg, thickness 0.02 mm, width 0.6 mm) can lift a weight of 1 kg. (F) SEM pictures of PFPE_1_–PCL_3_ under different strains. (G) 2D SAXS scattering patters of PFPE_1_–PCL_3_ under stretching demonstrating strain-induced orientation with increasing strain.

In addition to its soft yet super-tough properties, PFPE_1_–PCL_3_ also exhibits muscle-like self-reinforcing properties through a repetitive mechanical-training process. During small stretches within Hooke's elastic deformation (Fig. 3A, 50%), the cyclic curves are similar to traditional elastomers that exhibit a tensile stress decline as the cyclic numbers increase due to the rupture of the dynamic bonds and physical networks [51–53]. However, when cycled at a slightly larger strain (Fig. 3B, 100%), the stress is enhanced marginally as the loading–unloading tensile test is going on. More intensive mechanical training (cycling at 300%, 400% and 500% for 300 cycles) imparted the elastomers with stronger mechanical properties. As depicted in Fig. 3C and Fig. S14A and B, the elastomers strengthened dramatically compared with small stretches. Furthermore, the mechanical strength displayed a larger growth as the training process increased from 50 to 300 cycles. However, increasing the number of cycles to 600 results in only marginal improvements, suggesting that 300 cycles are adequate for the training (Fig. S14A and C).

Tensile tests were employed to assess the mechanical properties of the elastomers after mechanical training. As detailed in Fig. 3D, the tensile stress after mechanical training for 300 cycles increased by about 1.5∼3-fold in comparison with the original PFPE_1_–PCL_3_ at different training strains. Upon training at 500% strain for 300 cycles, the polymer (denoted as PFPE_1_–PCL_3_–500%–300 C) exhibited unprecedented mechanical strength and toughness, with an ultimate tensile strength of ∼228 MPa, yet it could still be stretched by 970%. As shown in Fig. 3E, PFPE_1_–PCL_3_–500%–300 C is capable of withstanding 200 000 times its own weight as an ultra-fine fiber (weight 5 mg, thickness 0.02 mm, width 0.6 mm). Additionally, the cyclic loading–unloading tensile tests at 50% strain show that the self-reinforcing PFPE_1_–PCL_3_ possesses low hysteresis (Fig. S14D), indicating excellent resilience. Moreover, the strengthened PFPE_1_–PCL_3_ remains stable at room temperature, with PFPE_1_–PCL_3_–100% showing no significant decline in mechanical properties after 7 days (Fig. S15).

To elucidate the mechanism behind the impressive mechanical performance and mechanical-training-driven self-reinforcing effect of PFPE_1_–PCL_3_, we characterized the structural evolution of PFPE_1_–PCL_3_ during stretching under different strains. As evidenced by using SEM, as shown in Fig. 3F(i)–(iv) and Fig. S16, before stretching, the elastomer showed a clustered, phase-separated and amorphous structure. Under a small strain (100%–300%), the elastomer gradually aligned into a curvy and slack structure. Increasing the strain (500%–700%) resulted in parallel and tightly aligned nanofibrillar architectures. Similar aligned nanofibrillar structures were observed by using polarized optical microscopy, as depicted in Fig. S17. Before deformation, the *in situ* small-angle SAXS displayed single isotropic scattering patters that stem from the amorphous and randomly distributed polymer chains and clusters (Fig. 3G(i)). Increasing the strain resulted in rapidly enhanced peak intensity and sharp streaks in the SAXS patterns before 300% (Fig. 3G(ii)), indicating that the initial amorphous and clustered polymer chains extended and oriented along the stretching direction. Further increasing the strain to 500% and 1200% led to sharper streaks (Fig. 3G(iii) and (iv)). We then availed XRD to qualitatively evaluate the variation in the oriented structure after training at different strains for 300 cycles (Fig. S18). The XRD peak intensity gradually increased upon mechanical training from 50% to 500% strain, indicating increased crystallization.

Based on these results, a conclusion can be obtained. The uniform phase separation and self-assembled structure of the prepared block copolymer PFPE_1_–PCL_3_ resulted in a clustered and amorphous structure, imparting mechanical softness, elasticity and high stretchability. Upon applying strain, the clustered polymer chains unfolded, straightened and reoriented toward the direction of loading, as manifested by the huge energy dissipation under large deformation (Fig. 2F). The PCL blocks were prohibited from forming stable inter/intra-chain lamellae by PFPE blocks before stretching. However, the PCL blocks easily escaped from the clusters and amorphous region to form new inter-chain microcrystalline domains along the driving direction as the polymer chains extended [43]. The microcrystalline and ordered nanostructures formed during stretching and mechanical training, which reinforced the elastomers and endowed the material with high strength, toughness and stretchability without sacrificing resilience. Thus, with the controlled hierarchical nanostructures, the soft yet ultra-strong elastomers had the required combination of super-high strength and mechanical-training-reinforced characteristics that can properly mimic skeletal muscles.

### Shape-memory property and application in artificial muscle actuation

**Figure 4.**
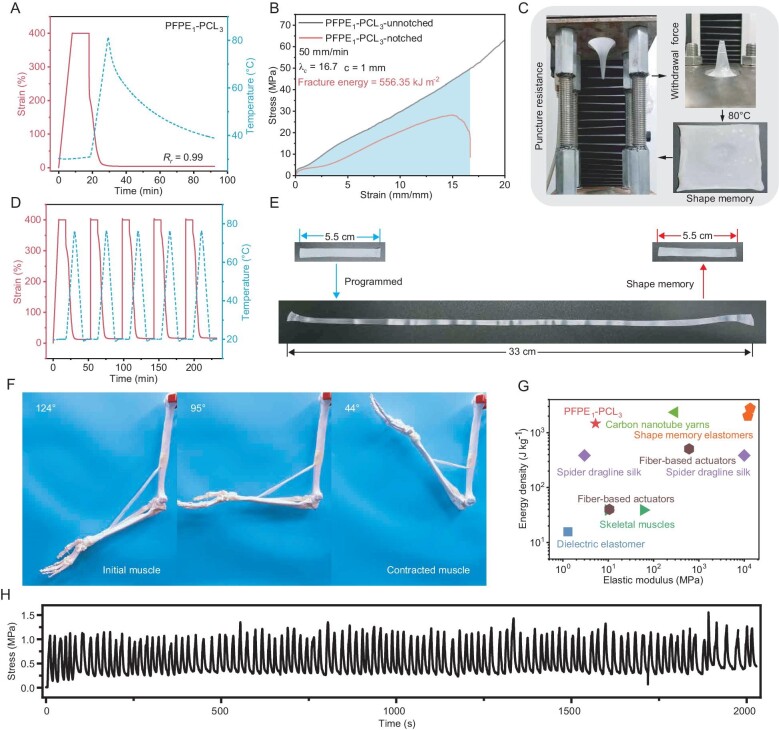
Shape-memory properties and application in artificial muscles. (A) Shape-memory program and recovery curves obtained by using DMA. (B) Stress–strain curves of the unnotched and notched samples. (C) Images displaying the puncture tests and the elastomer recovery to initial state upon heating. (D) Multiple program and actuation behaviors of PFPE_1_–PCL_3_ tested by using DMA. (E) Photographs of PFPE_1_–PCL_3_ at the initial state, stretched to 600% and contracted by heat stimuli. (F) Pre-stretched PFPE_1_–PCL_3_ artificial muscle (1.5 g) actuates a 350-g, 70-cm life-sized upper-limb model when heated. (G) Literature comparison of the actuation performance based on energy density and elastic modulus between different kinds of actuators. (H) Reversible actuation performance of the pre-stretched PFPE_1_–PCL_3_ at a fixed length under a repetitive heating–cooling process.

The crystalline state is fairly stable once formed, so the polymer can be fixed upon stretching for >30 days without returning to its original dimensions (Fig. S19A). However, upon melting and cooling, the polymer turns back into the amorphous state. The reversible crystallizing-and-melting process leads to the shape-memory behavior (Fig. S19A). When successively stretched, the energy dissipation capacity, determined from the hysteresis area, is significantly lower during the second cycle (3.15 MJ m^−3^) than during the first cycle (16.28 MJ m^−3^). Nonetheless, this capacity can be fully restored with thermal stimulation (Fig. S19B), demonstrating full recovery to the original state via shape memory. Dynamic mechanical analysis (DMA) quantitatively confirmed a complete shape recovery (*R*_r_ = 0.99) when the temperature was increased to 80°C (Fig. 4A). Therefore, our polymer might be able to function as an artificial muscle.

Natural muscles not only demonstrate excellent stimuli-responsive properties, but also remarkable resistance to external damage [33,37]. To verify the suitability of the prepared artificial muscles for skeletal muscle replacement in large mammals suffering from VML, we performed tear and puncture resistance tests to evaluate their durability and structural integrity. As evidenced in Fig. 4B and Fig. S20, the PFPE_1_–PCL_3_ with a notch (width of 1 mm) exhibited an excellent tear resistance property. Notably, no evident propagation of the crack was observed, even at a strain of 1400%. The pre-notched PFPE_1_–PCL_3_ demonstrated ultimate stress and strain as high as 28.17 MPa and 1670%, respectively. This benefit arises from the rapidly oriented nanofibrils that are aligned perpendicular to the crack, which promotes notch passivation and improves tear resistance. The fracture energy was calculated to be 556.35 kJ m^−2^, which is comparable to the highest-toughness elastomers reported in the literature [54,55]. Moreover, a strip of thin film of PFPE_1_–PCL_3_ (weight of 0.2 g, thickness of 0.15 mm) with a notch could withstand a weight of 2.6 kg without cracking, demonstrating its ultra-strong mechanical properties (Fig. S20B).

Puncture resistance tests were conducted with a PFPE_1_–PCL_3_ film of 0.4 mm in thickness. As shown in Fig. 4C, Fig. S21 and Movie S1, PFPE_1_–PCL_3_ showed a high needle displacement of ∼6 cm and could withstand a force of ∼28.5 N with such a thin film, resulting a puncture energy of 766.78 mJ. Furthermore, PFPE_1_–PCL_3_ offers a unique property that distinguishes it from previous puncture-resistant materials—the capability of shape memory. As depicted in Fig. 4C and Movie S2, the bulk film sustained a ‘conical shape’ as the needle was removed. Upon heating to 80°C, the damaged sample fully recovered to its original state within a few seconds. Successive cyclic puncture resistance tests were performed to estimate the mechanical rehabilitation ability after the reshaped sample was relaxed for 30 min at room temperature. As seen in Fig. S21, PFPE_1_–PCL_3_ could restore its puncture resistance ability to >90% after five rounds of the cyclic puncture test.

Bionic artificial muscle materials that are capable of generating considerable force and motion when exposed to external stimuli (such as light and heat) have aroused considerable interest and are hailed as a potential solution for treating VML (especially for amputees). We therefore carried out experiments to testify the potential of the elastomers for artificial muscle actuators. DMA was employed to evaluated the repeatable program–actuate ability of the elastomers under multiple heating–cooling processes. PFPE_1_–PCL_3_ demonstrated a minimal hysteresis (*R*_r_ = 0.99) at a large strain of 400% even after five cycles (Fig. 4D) and yielding an actuation stress of 2.8 MPa (Fig. S22). As seen in Fig. 4E, a strip of PFPE_1_–PCL_3_ fixed at 600% could fully recover to its original state with an ultra-long actuating distance of 27.5 cm. Several weight-lifting tests were conducted to assess the work capacity and potential of PFPE_1_–PCL_3_ to be applied as a soft, robust and large-strain actuator. As demonstrated in Fig. S23A and Movie S3, a pre-programmed thin PFPE_1_–PCL_3_ film (4 mg) could lift 5000 times its own weight by 2.9 cm upon heating in a second with a high energy density of 1450 J kg^−1^, which is >37 times the energy density of skeletal muscle (39 J kg^−1^) [56]. By virtue of the high extensibility, the PFPE_1_–PCL_3_ elastomers displayed an ultra-long driving distance, as a film of 40 mg could lift a weight of 20.0 g by 19.0 cm (corresponding to an energy density of 950 J kg^−1^), which is the maximum driving distance reported in the literature (Figs S23B and S24, Table S5 and Movie S4). We then demonstrated its potential in artificial muscles by actuating a robotics arm to lift weights via a pre-stretched film. A 20-mg elastomer can contract to lift a weight of 50 g rapidly with an actuation angle between the forearm and humerus of 102° (Fig. S23C and Movie S5). To demonstrate the potential of PFPE_1_–PCL_3_ as a prosthetic actuator, a pre-stretched PFPE_1_–PCL_3_ artificial muscle was used to drive a life-sized upper-limb model. When heated, it contracted the arm from 124° to 44° (the angle is defined as the angle between the upper arm and the forearm), covering the range of daily grasping movements (Fig. 4F). In contrast to traditional high-modulus shape-memory polymers, PFPE_1_–PCL_3_ exhibited simple and high-efficiency programmable routes, benefitting from the mechanical softness properties (Movie S6). Finally, we characterized the reversible actuation behaviors via controlling the melting process of the crystalline domains during the multiple heating–cooling cycles. The arm that lifted a 50-g weight displayed reversible action motions with an angle range of >15° (Fig. S23D and Movie S7). By using a fixed action length, the reversible actuation force was determined by carefully controlling the heating–cooling process. The PFPE_1_–PCL_3_ consistently and quickly generated contractile force for >120 cycles without any performance deficiency (Fig. 4H), achieving reliable and reversible actuation that was akin to that of mammalian muscles. Combining the merits of softness, long driving distance, high energy density, fast responsibility and reversible actuation, PFPE_1_–PCL_3_ exhibited a huge advantage compared with previous works (Fig. 4G, Fig. S24 and Table S5). Moreover, benefitting from the linear polymer chain structures and physical cross links, PFPE_1_–PCL_3_ demonstrated satisfactory recyclability and reprocessability under specific conditions. After five cycles, the recycled material could essentially maintain its mechanical properties and shape-memory characteristics (Fig. S25).

### Application in *in vitro* and *in vivo* VML model

**Figure 5.**
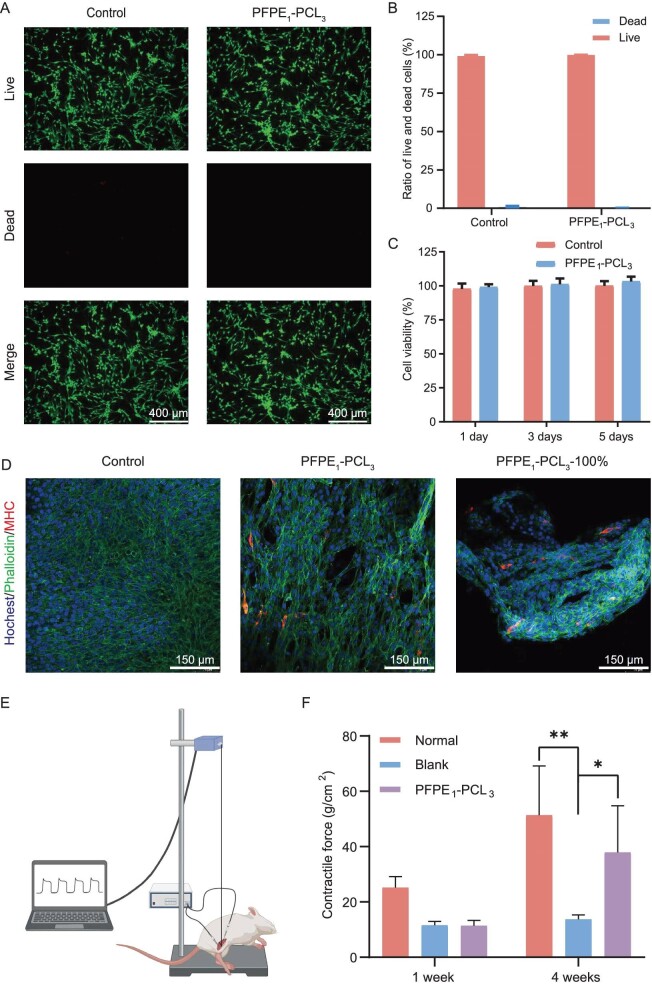
Cell cytotoxicity, proliferation and differentiation of PFPE_1_–PCL_3_. (A) Live/dead staining of C2C12 cells cultured with PFPE_1_–PCL_3_ for 24 h (scale bar = 400 μm) and (B) statistical analysis of the ratio of live cells and dead cells. (C) Cell viability of C2C12 cells cultured with PFPE_1_–PCL_3_ for 1, 3 and 5 days detected by using CCK-8 assay. (D) MHC staining (red) and cytoskeleton staining by phalloidin (green) of C2C12 cells cultured on different samples for 7 days (scale bar = 150 μm). Nuclei were stained blue by using Hoechst. All data are presented as mean ± SD, *n* = 4. (E) Scheme of electromyography test was created using BioRender (biorender.com) and (F) statistical analysis of contractile force of the regenerative muscles at Week 1 and 4 of the normal, blank and PFPE_1_–PCL_3_ groups. *P < 0 .05; **P < 0.01, compared with the blank group.

To explore the application of biomimetic artificial muscle materials in VML models, we validated the potential of PFPE_1_–PCL_3_ as a muscle replacement material through *in vitro* and *in vivo* experiments. Firstly, live/dead staining that was performed after 24 h of co-culture with C2C12 myoblasts (Fig. 5A and B) indicated no significant differences from the control group, with no evident dead cells (red). Furthermore, we assessed the biocompatibility of PFPE_1_–PCL_3_ by using the CCK-8 assay. The results showed no significant differences compared with the control group after co-culturing with C2C12 myoblasts for 1, 3 and 5 days (Fig. 5C), consistently with the C2C12 myoblasts results. These findings demonstrate that PFPE_1_–PCL_3_ possesses good biocompatibility and is non-cytotoxic. Next, we investigated the effect of PFPE_1_–PCL_3_ and PFPE_1_–PCL_3_–100% (samples after 100% stretching orientation) on the differentiation of muscle satellite cells, to determine whether it can promote muscle growth and replicate the underlying mechanisms. After it was co-cultured with C2C12 myoblasts for 7 days, myosin heavy chain (MHC) staining was conducted (Fig. 5D and Fig. S26). The results revealed elongated myotubes in both the PFPE_1_–PCL_3_ and PFPE_1_–PCL_3_–100% groups. In the PFPE_1_–PCL_3_–100% group, the myotubes were oriented along the stretching direction of the material, indicating that the directional alignment of PFPE_1_–PCL_3_ after 100% stretching promotes the directional growth and myogenic differentiation of C2C12 cells.

Based on these results, we implanted PFPE_1_–PCL_3_ into a rat VML model (Fig. S27) to evaluate its potential as a substitute for functional muscle loss. As shown in Movie S8, the superior elasticity and softness of PFPE_1_–PCL_3_ enabled the implanted material to facilitate the flexion and extension of the residual muscle. Moreover, due to the mechanical robustness and strain alignment properties of PFPE_1_–PCL_3_ (Fig. 3F), the mice could maintain their routine activities after scaffold implantation, without the muscle atrophy or joint function impairment that are typically caused by traditional surgical sutures with plaster immobilization. During the voluntary movements of the mice, the scaffold stretched and aligned, providing mechanical stimulation to the muscle tissue and promoting muscle fiber growth [6]. This mechanism may contribute to the effectiveness of PFPE_1_–PCL_3_ in enhancing muscle tissue regeneration. *In vivo* electrical stimulation was performed at 1 and 4 weeks post-implantation to assess muscle function and the muscle contraction force was measured (Fig. 5E and F). The results indicated no significant difference in muscle contraction force between the PFPE_1_–PCL_3_ group and the blank group at 1 week post-implantation. At 4 weeks post-implantation, the contraction force of the PFPE_1_–PCL_3_ group was significantly higher than that of the blank group and approached that of the normal group, suggesting that PFPE_1_–PCL_3_ may promote muscle fiber growth. Compared with the scaffold materials that are reported in the current literature, our material not only significantly enhances the muscle growth rate, but also exhibits high mechanical strength and multifunctional properties (Table S6).

**Figure 6.**
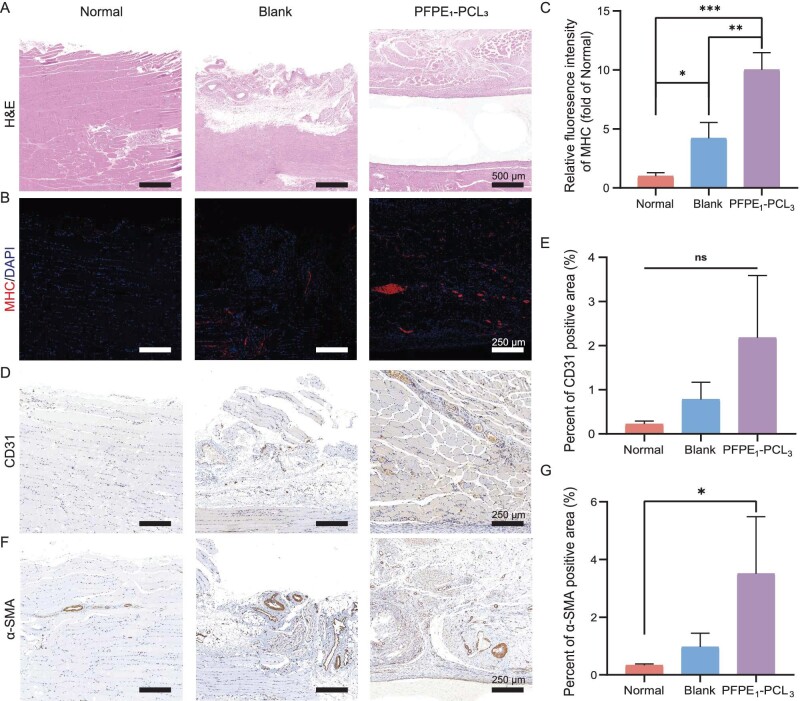
Promotion of angiogenesis and muscle repair 4 weeks after PFPE_1_–PCL_3_ implantation *in vivo*. (A) Representative images of HE staining of TA muscle treated in different groups at Week 4 post-injury (scale bar = 500 μm). (B) Representative images of immunofluorescence staining of TA remodeled muscle treated in different groups with MHC stained as red at Week 4 post-injury (scale bar = 250 μm). (C) Quantitative analysis of immunofluorescence staining of MHC. (D) Representative images of CD31 staining of TA muscle treated in different groups at Week 4 post-injury (scale bar = 250 μm). (E) Quantitative analysis of the expression of CD31. (F) Representative images of α-SMA staining of TA muscle treated in different groups at Week 4 post-injury (scale bar = 250 μm). (G) Quantitative analysis of the expression of α-SMA. All data are presented as mean ± SD. ns *P* > 0.05; *P < 0.05; **P < 0.01; ***P < 0.001. *n* = 4.

To verify this hypothesis, we extracted the tibialis anterior (TA) muscles at 1 and 4 weeks post-implantation for HE staining (Fig. 6A and Fig. S28A). The results showed localized inflammation and muscle loss in the blank group at both 1 and 4 weeks post-surgery, with muscle fibrosis observed at 4 weeks. In the PFPE_1_–PCL_3_ group, the scaffold was observable and muscle tissue grew along the scaffold. At 4 weeks post-surgery, the regenerated muscle exhibited good structure and morphology. Additionally, MHC staining indicated more muscle fibers in the PFPE_1_–PCL_3_ group (Fig. 6B and C, and Fig. S28B and C), further supporting that PFPE_1_–PCL_3_ promotes muscle fiber growth. To evaluate the pro-angiogenic performance of PFPE_1_–PCL_3_, we conducted CD31 and α-smooth muscle actin (α-SMA) staining on muscle tissue. At 1 week post-surgery, the CD31 staining results showed no significant difference in the PFPE_1_–PCL_3_ group compared with the blank group (Fig. S28D and E). Although there was an increase in the CD31+ vessel density in the PFPE_1_–PCL_3_ group at 4 weeks post-surgery, it was still not significantly different from that of the blank group, indicating some improvement in vascularization (Fig. 6D and E). These results suggest a partial improvement in angiogenesis. However, unlike the CD31 staining results, at 1 week post-surgery, the α-SMA+ vessel density in the PFPE_1_–PCL_3_ group was significantly higher than that in the blank group (Fig. S28F and G). Additionally, at 4 weeks post-surgery, the trend was similar to that of CD31+, with an increase in expression, but the difference was not significant (Fig. 6F and G). Therefore, PFPE_1_–PCL_3_ has the potential to promote angiogenesis, which also aids in muscle regeneration.

## CONCLUSION

This study explores the treatment of VML through the development of multifunctional artificial muscles: PFPE_1_–PCL_3_. The synthesized materials exhibit essential mechanical properties including low elastic modulus (5.27 ± 0.05 MPa), high tensile strength (72.67 ± 3.19 MPa) and exceptional toughness (742.02 ± 23.98 MJ m^−3^), which are crucial for long-term tissue regeneration and prosthetic actuation. The superior biocompatibility and soft mechanical profile of PFPE_1_–PCL_3_ facilitate its integration into the host tissue, which is crucial for successful long-term outcomes. Additionally, PFPE_1_–PCL_3_ showcases superior actuation performance and mechanical strengthening characteristics. These properties ensure that the artificial muscles not only promote skeletal muscle-healing processes, but also address the major limitations of the current scaffold-based approaches. Its robust actuation capabilities suggest potential applications in prosthetic devices, significantly expanding the clinical use of the scaffolds. The artificial muscles that were developed in this study not only meet the immediate needs of patients who are suffering from VML, but also offer a promising pathway for developing next-generation biomaterials for muscle repair and prosthetic applications. This research provides a foundation for future technological advances and opens up new avenues for improving the quality of life for millions who are affected by severe muscle loss.

## MATERIALS AND METHODS

### Materials

Polycaprolactone diol (*M_n_* = 2000 g mol^−1^) was purchased from Aladdin. Fluorolink E10H (PFPE–OH, *M_n_* ≈ 1800 g mol^−1^) was bought from Alpha Aesar. These reagents were dried under a vacuum at 90°C for 12 h and then stored in a dryer with a N_2_ atmosphere. HDI (99%) and dibutyltin dilaurate (DBTDL, 95%) were purchased from Aladdin. *N,N*-dimethylacetamide (DMAc, 99.8%, extra dry, with molecular sieves) and dimethylbenzene (99.8%, extra dry, with molecular sieves) were obtained from Energy Chemical.

### Synthesis of polymers and preparation of the films

The typical synthesis routes of the PFPE*_x_*–PCL*_y_* series elastomers are shown in Fig. S2 and the detailed preparation procedure was as follows. Taking PFPE_1_–PCL_1_ as an example, a two-neck bottom flask (100 mL) equipped with a Schlenk system and magnetic stirrers was heated at 85°C under a vacuum for 30 min and then under N_2_ to remove moisture. The mixture of PFPE–OH (1 mmol), DBTDL (0.01 g), HDI (2 mmol) and xylene (3 mL) was added into the flask via a syringe and heated at 85°C for 1 h, followed by the addition of a solution of PCL–OH (1 mmol) and DMAc (6 mL). More DMAc (∼25 mL) would be needed due to the rapidly increased viscosity. The whole reaction system was heated at 85°C for 15 h under stirring and a N_2_ atmosphere. Finally, the resulting highly viscous polymer solution was poured into a polypropylene mold to remove the solvent at room temperature for >72 h and then decanted into an oven for drying at 70°C for at least 24 h to obtain the test samples.

## ETHICAL STATEMENTS

This study was performed in accordance with the recommendations in the Guide for the Care and Use of Laboratory Animals and relevant Chinese laws and regulations. The protocol was approved by the Institutional Animal Care and Use Committee (IACUC) of Shanghai Jiao Tong University and the Animal Protocol number is A2024228.

## Supplementary Material

nwae422_Supplemental_Files
