# Supplementary material for: A soft, ultra-tough and multifunctional artificial muscle for volumetric muscle loss treatment
Source: Natl Sci Rev. 2024 Nov 22;12(2):nwae422. doi: 10.1093/nsr/nwae422 (PMC11737398; doi:10.1093/nsr/nwae422)
Supplement: nwae422_Supplemental_Files [file nwae422_supplemental_files.zip › Supplementary data.pdf]

Supporting Information

A soft, ultra-tough and multifunctional artificial muscle for volumetric muscle loss treatment

Peng-Fei Qiu[1,†], Lei Qiang[2,†], Weiqing Kong[3], Fang-Zhou Wang[1], Hong-Qin Wang[1], Ke-Xin Hou[1], Yihao Liu[4], Cheng-Hui Li[1,*] and Pengfei Zheng[2,*]

[1]State Key Laboratory of Coordination Chemistry, School of Chemistry and Chemical Engineering, Collaborative Innovation Center of Advanced Microstructures, Nanjing University, Nanjing 210023, China;

[2]Department of Orthopaedic Surgery, Children's Hospital of Nanjing Medical University, Nanjing 210004, China;

[3]Department of Orthopedic Surgery, Xuzhou Central Hospital, Xuzhou Clinical School of Xuzhou Medical University, Xuzhou 221009, China;

[4]Shanghai Key Laboratory of Orthopedic Implant, Department of Orthopedic Surgery, Shanghai Ninth People's Hospital, Shanghai Jiao Tong University School of Medicine, Shanghai 200011, China

*__Corresponding authors__. E-mails: chli@nju.edu.cn; zhengpengfei@njmu.edu.cn

[†]Equally contributed to this work.

1. Experimental Section

1.1 General characterization: FT-IR spectra were obtained from a Bruker Tensor 27. Differential scanning calorimetry (DSC) tests were conducted on a Mettler DSC 1 system under N_2 atmosphere range from -80 ℃ to 110 ℃, and the heating/cooling rate was set as 10 K min^{-1}. Thermal gravimetric analysis (TGA) experiments were performed on the STA 449C instrument in N_2 atmosphere from 30 ℃ to 600 ℃ with a heating rate of 10 K min^{-1}. X-ray diffraction (XRD) data were recorded on a Bruker Advance D8 (40 kV, 40 mA) diffractometer with Cu Kα radiation (λ = 1.54056 Å) at room temperature with a scan speed of 0.2 °/step. Gel permeation chromatography (GPC, Agilent 1260) was employed to measure the polymers' molecular weights with *N*, *N*-Dimethylformamide (DMF) as the mobile phase. Polarized optical microscopy (POM) images were obtained on a Mshot MP41 at room temperature. Scanning electron microscopy (SEM) films were imaged on a Hitachi S-4800 SEM at 10 kV. Transmission electron microscopy (TEM) images were obtained from a Tecnai G1 F30 S-TWIN with the samples were freezing ultrathin-sectioned. Small-Angle X-ray Scattering (SAXS) were performed using a Cu Kα source (0.154 nm) Nanostar U (BRUKER, Germany) instrument fitted with collimation optics and a 2D X-ray detector (Vantec 2000). The scattering wavevector, q, was calibrated by using a standard sample of powder silver behenate. The samples were placed and measured under vacuum. The scattering images were captured with a Vantec 2000 detector. The sample-to-detector distance was 1050 mm.

1.2 Tensile tests: All of the tensile tests were performed on an Instron 3343 instrument with a stretching speed of 50 mm min^{-1} at room temperature without further explanation. For each tensile test, dumbbell-shaped samples were tailored with an active region of 8 mm × 2 mm × 0.15 mm and at least five individual tests were conducted for different kinds polymer films. Cyclic tensile tests were employed with a loading/unloading process at different strain (without relaxed waiting time) with a stretching speeding of 50 mm min^{-1} at room temperature. Step cyclic loading-unloading tests were conducted by set the strain from 100%

to 1500% with a stretching speeding of 50 mm min^{-1} under room temperature with different samples.

Tensile toughness (τ) and hysteresis area were calculated by integrating the area under strain-stress curves and the area of each cyclic strain-stress curves (equation 1), the stretching speed of cyclic tests was set at 50 mm min^{-1}.

$$\tau = \int_{\varepsilon=0}^{\varepsilon=\varepsilon_{max}} \sigma \, d\varepsilon \qquad (1)$$

1.3 Puncture resistance tests: The puncture tests were conducted by Instron 3343 instrument by setting the speed of the needle of 50 mm min^{-1} with a sample-holding apparatus and a needle (tip radius, R = 1.59 mm), the thickness of the sample is 0.4 mm. For cyclic puncture tests, tests were paused until the puncture distance approach the max displacement to protect the sample from broken and withdraw the force, then the samples were heat at 80 ℃ then release for 30 min at room temperature for next puncture test.

1.4 Tear resistance tests: The tear resistance tests were employed to calculate the fracture energy (G_c) (equation 3) by tensile tests with the single-edge notched sample (width of 6 mm, thickness of 0.15 mm, length of 8 mm, notched length is 1 mm), the stretching speed was setting as 50 mm min^{-1} [1].

$$G_c = \frac{6wc}{\sqrt{\lambda_c}} \qquad (3)$$

Where c denoted as the length of the notch, λ_c denoted as the strain at the break of the notched sample, w is the strain energy of the unnotched sample calculated by integrating the strain-stress curves until ε_c ($\varepsilon_c = \lambda_c - 1$).

1.5 Shape memory tests: Shape memory tests were conducted by dynamic mechanical analysis on a DMA Q800 instrument. The shape program of the samples was performed into DMA at 30 ℃, with the sample initially deformed to 400% strain at 50% strain per minute, and held for 10 min to fix the deformation, then the force was released to 0.001 N, increase the temperature to 80 ℃ at 5 ℃ min^{-1}. Actuation stress measurements were performed with a

pre-programmed sample (600%) held under constant length, then ramped the temperature to 80 ℃ at 5 ℃ min^{-1}.

1.6 Measurement of the reversible actuation stress: The reversible actuation performance of the artificial muscles was measure by a AILIGU DIGITAL DYNAMOMETER ZP-5N instrument with a 15-cm-long pre-programmed sample. Carefully controlling the heating-cooling process while maintaining the distance between the two fixtures constant, then converting the recorded force to stress by using the initial cross-section area of the samples.

1.7 Recycle experiments: The $PFPE_1$-PCL_3 (~2 g) were cut into pieces and dissolved into solvent of DMAc at 70 ℃, then poured into a mold, the solvent evaporated at room temperature for more than 72 h and then at 70 ℃ for another 24 h to get a film.

2. *In Vitro* Study

2.1 Cell Culture: The C2C12 mouse myoblast cell line was obtained from the Cell Bank of the Chinese Academy of Sciences. The complete culture medium was Dulbecco's modified Eagle medium (DMEM, Gibco) supplemented with 10% fetal bovine serum (FBS, Gibco) and 1% penicillin/streptomycin (P/S, Hyclone). Cells were cultured in an incubator at 37 ℃ with 5% CO2. When cell confluence reached approximately 80%, cells were digested with 0.25% trypsin-EDTA (Gibco) and passaged at a 1:2 ratio.

2.2 Material Grouping and Sterilization: $PFPE_1$-PCL_3 was stretched to 100%, referred to as the $PFPE_1$-PCL_3-100% group, while the unstretched group was referred to as the $PFPE_1$-PCL_3 group. The materials were cut into 1 cm × 1 cm and 1 cm × 0.3 cm squares for cell culture and in vivo study. Then they were soaked in 75% ethanol for 1 hour, followed by rinsing three times with sterile PBS, each rinse lasting 10 minutes.

2.3 Cytotoxicity and Proliferation: Cell proliferation was assessed using the CCK-8 assay. Sterile materials were placed in a 48-well plate, and C2C12 cells were seeded at 5000 cells/well on both blank wells and material samples. At days 1, 3, and 5, CCK-8 solution was

added, incubated at 37 ℃ for 1 hour, and absorbance was measured at 450 nm using a microplate reader (BioTek). Cytotoxicity of the materials was evaluated using a Live/Dead staining kit (YEASEN). After 24 hours of co-culture with cells, the medium was removed, live/dead staining solution was added, and the samples were incubated at 37 ℃ for 30 minutes. Images were captured using a confocal microscope (Leica).

2.4 Immunofluorescence Staining: After 7 days of culture for each group, the medium was removed and the samples were washed three times with PBS, each wash lasting 5 minutes. Cells were fixed with 4% paraformaldehyde (Servicebio) at room temperature for 15 minutes. Cells were permeabilized in PBS containing 1% Triton X-100 for 5 minutes, then blocked with 5% BSA solution at room temperature for 30 minutes. Primary antibody against MHC (3.228 μg/ml, Abcam) was added and incubated at 4 ℃ for 12 hours, followed by the addition of secondary antibody (1:1000, Abcam). The cytoskeleton was stained with TRITC-Phalloidin (1:200, MKbio) at room temperature for 15 minutes, and nuclei were stained with Hoechst (Solarbio) at room temperature for 5 minutes. Stained samples were photographed using a confocal microscope (Leica).

3. *In Vivo* Study

3.1 VML Model Establishment: Twenty-four male SD rats (weighing 200 ± 20 g) were randomly divided into three groups: normal group (no surgery), blank group (surgery without implantation), and $PFPE_1$-PCL_3 group. After anesthetizing the rats with isoflurane, they were placed in a supine position. The skin and deep fascia over the TA muscle were longitudinally incised to expose the TA muscle, and approximately 30% of the TA muscle was excised. $PFPE_1$-PCL_3 was sutured to the ends of the residual muscle. The deep fascia and skin were sutured and disinfected with iodine. The rats were euthanized at 1 week and 4 weeks post-implantation. The protocol was approved by the Institutional Animal Care and Use Committee (IACUC) of Shanghai Jiao Tong University, the Animal Protocol number is A2024228. TA muscles were harvested, embedded in paraffin, sectioned, and subjected to H&E staining, CD31 and α-SMA immunohistochemical staining, and MHC immunofluorescence staining.

3.2 Functional Assessment: The contractility of the TA muscles was assessed at 1 week and 4 weeks post-surgery. As previously described [2], rats were anesthetized and placed in a supine position on the testing platform, with the legs positioned correctly and the foot and knee joint fixed to ensure isometric muscle contraction. Electrodes were placed subcutaneously in the TA muscle area and secured. Electrodes were adjusted until a regularly shaped tetanic curve was obtained. At least five tetanic contraction measurements were recorded for each group, and the length and weight of the TA muscle were measured. Peak tetanic force was selected for downstream analysis.

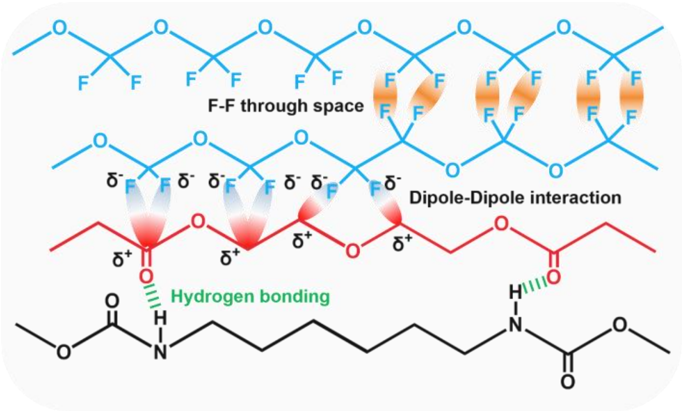

Fig. S1. Schematic illustration of the elastomer structure.

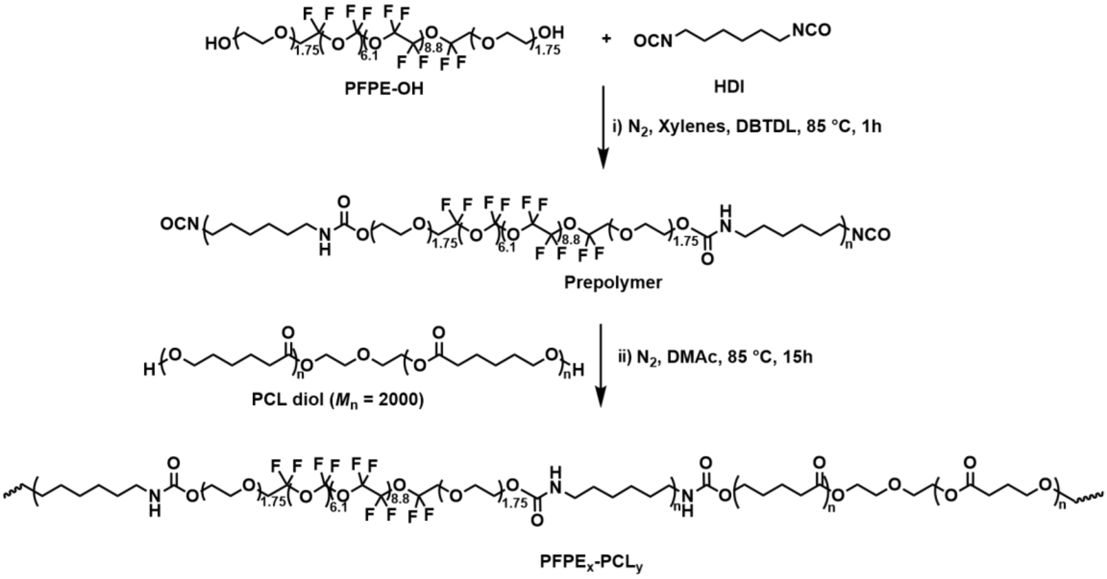

Fig. S2. Synthetic routes of the $PFPE_x$-PCL_y series elastomers.

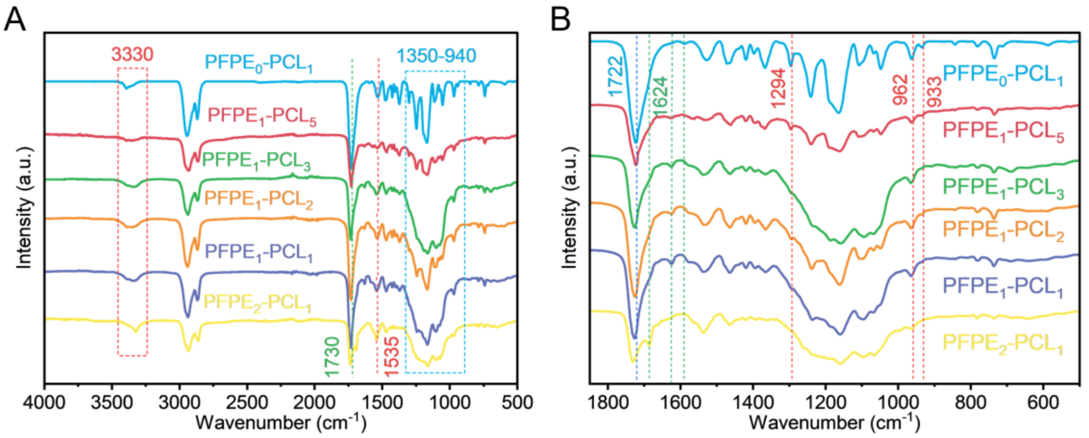

Fig. S3. FT-IR curves of the $PFPE_x$-PCL_y series elastomers. As the N-H stretching and bending vibration absorption peaks appeared at 1535 cm^{-1} and 3330 cm^{-1}, while the peaks at 3340 cm^{-1} and 2270 cm^{-1} which belong to the stretching vibration of the hydroxy group and isocyanate group are disappeared. The bands at 1350 - 940 cm^{-1} can be attributed to the stretching vibrations of C-F and CF_2 bonds. As depicted in Fig. S3B, $PFPE_0$-PCL_1 showed strong absorbance at 933 cm^{-1}, 962 cm^{-1}, and 1294 cm^{-1}, corresponding to the backbone C-C and C-O-C stretching vibrations in the crystalline [3, 4]. The intensity of these peaks decreased or even vanished with an increasing ratio of PFPE. The C=O peaks gradually shifted from 1722 cm^{-1} ($PFPE_0$-PCL_1) to 1732 cm^{-1} ($PFPE_2$-PCL_1), demonstrating that the introduction of PFPE could inhibit the crystallization of PCL [3]. The peaks at 1624 cm^{-1} can be ascribed to synergistic inter/intra-molecular interactions between PFPE and PCL chain segments [5,6], which were not observed in $PFPE_0$-PCL_1 and $PFPE_2$-PCL_1. Similar peaks were observed at 1587 cm^{-1} for $PFPE_0$-PCL_1 and 1684 cm^{-1} for $PFPE_2$-PCL_1. This difference likely originates from the variations in polymer chain entanglements. $PFPE_0$-PCL_1 easily forms tightly packed chains and lamellae due to its relatively simple and regular chain architecture. However, significant hindrance from the blocked PFPE segments and the large CF_2 groups exists in $PFPE_1$-PCL_5 to $PFPE_2$-PCL_1, resulting in loosely and irregularly packed polymer chain architecture. Additionally, the shape of peaks between 1335 cm^{-1} and 1000 cm^{-1} changed with increasing PFPE content. These changes were attributed to the coupled interactions among C-F, CF_2, C-C, C-O-C, C-C-H, and O-C-H [3, 6].

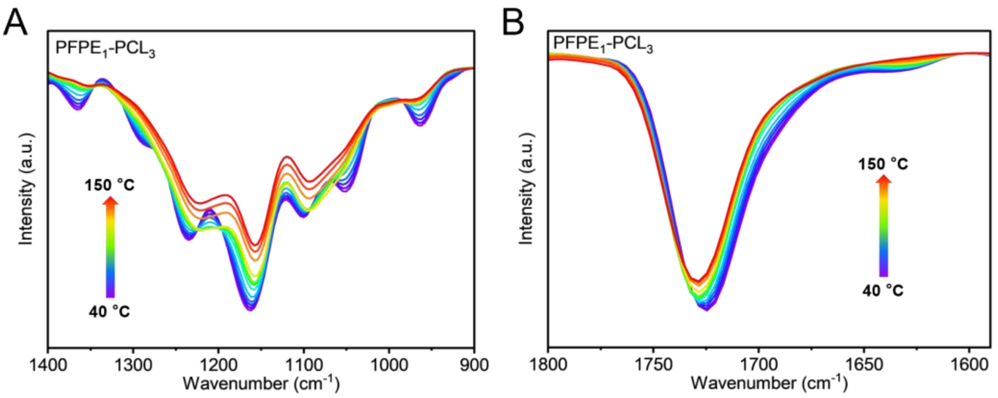

Fig. S4. Temperature-dependent FT-IR spectra of $PFPE_1$-PCL_3. Upon heating, the intensity of peaks around 1730 cm^{-1} and in the range of 1350 - 940 cm^{-1} decreased and shift to high wavenumber, indicate the decline of dipole-dipole force between PFPE and PCL segments due to that the accelerated thermal motion disrupted the orderly arrangement of polymer chains.

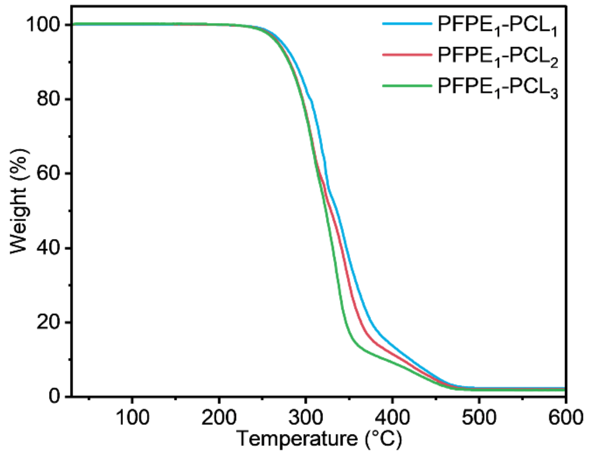

Fig. S5. TGA curves of the $PFPE_x$-PCL_y elastomers.

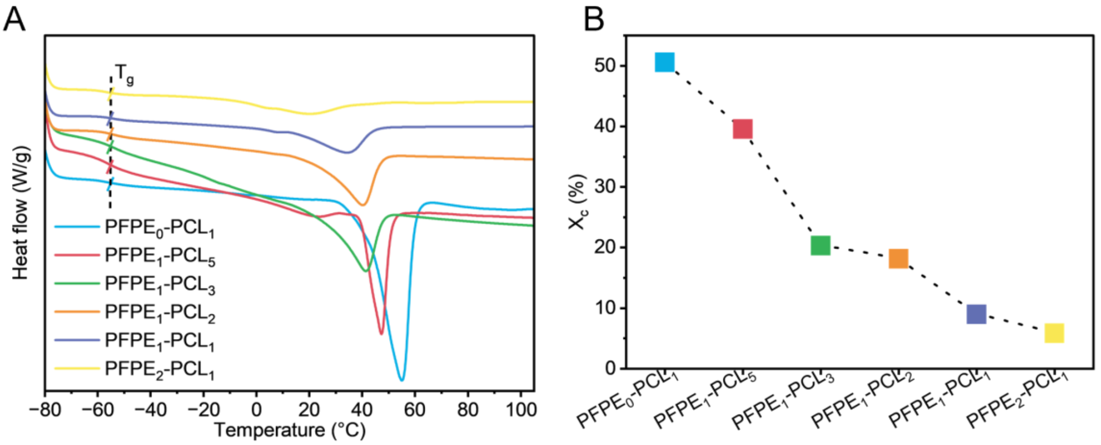

Fig. S6. The DSC curve and the corresponding crystallinity of PFPE$_x$-PCL$_y$.

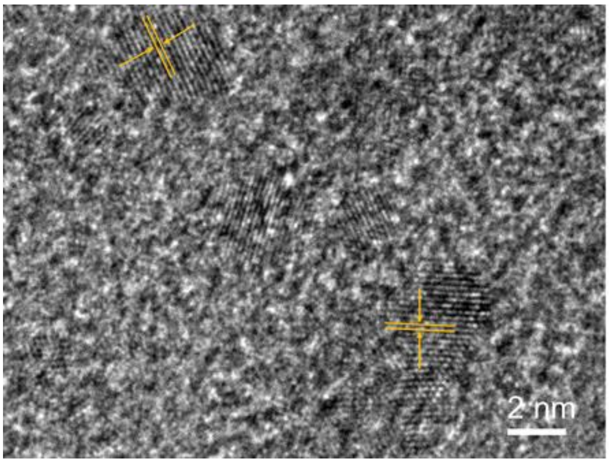

Fig. S7. TEM image of PFPE$_0$-PCL$_1$.

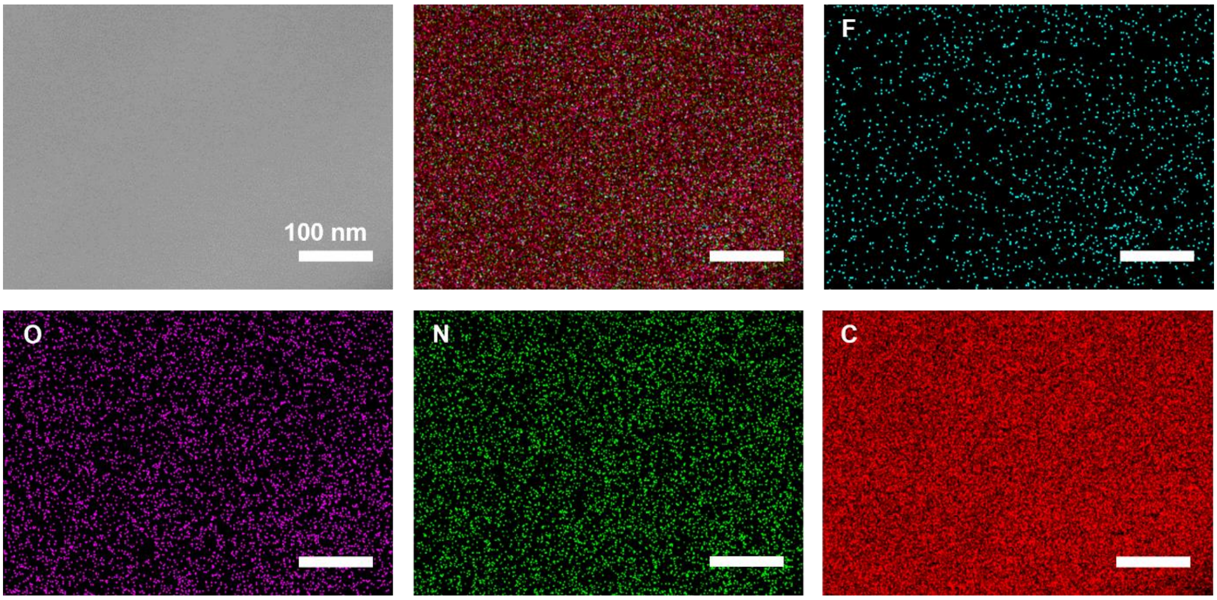

Fig. S8. Energy Dispersive Spectroscopy (EDS) image of $PFPE_1$-PCL_3 from TEM.

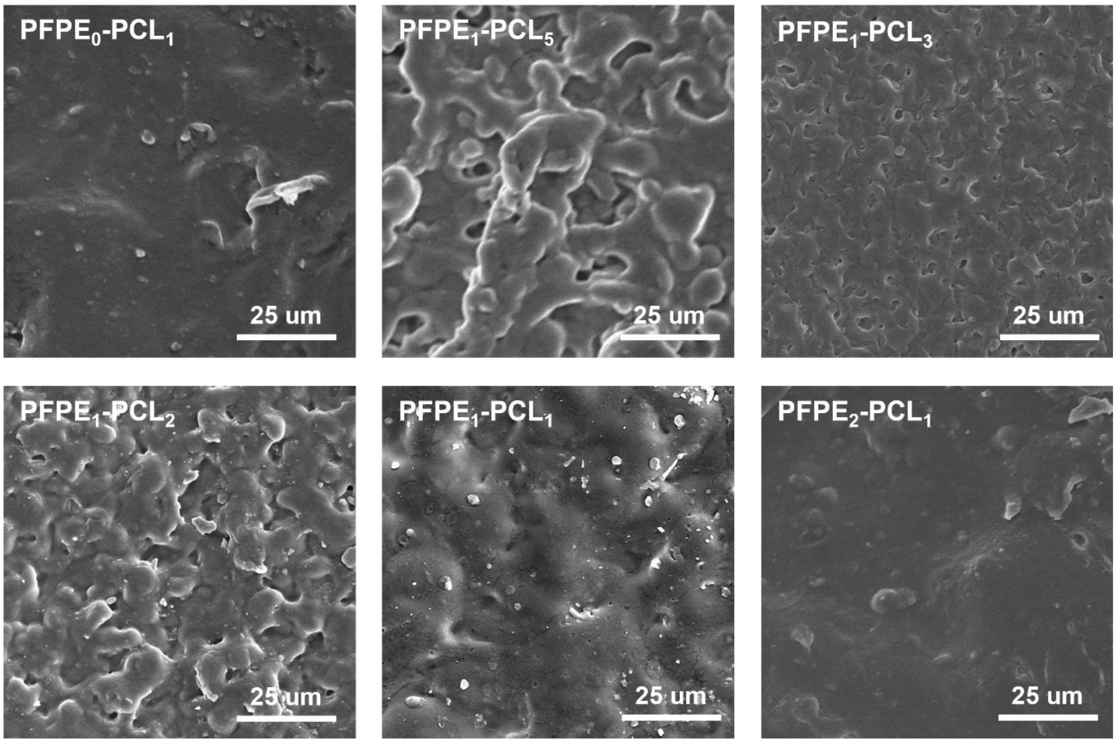

Fig. S9. SEM images of PFPE$_x$-PCL$_y$.

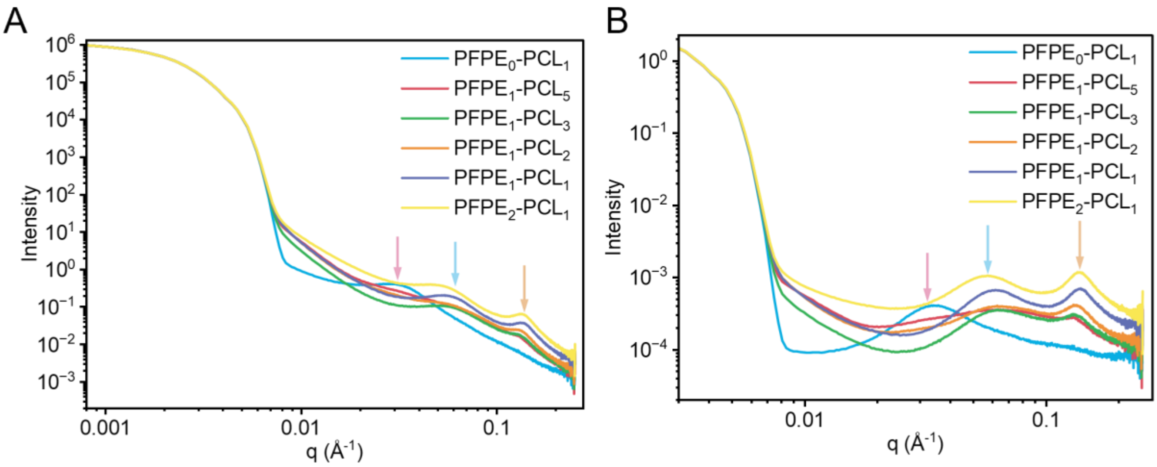

Fig. S10. SAXS patterns of the PFPE$_x$-PCL$_y$.

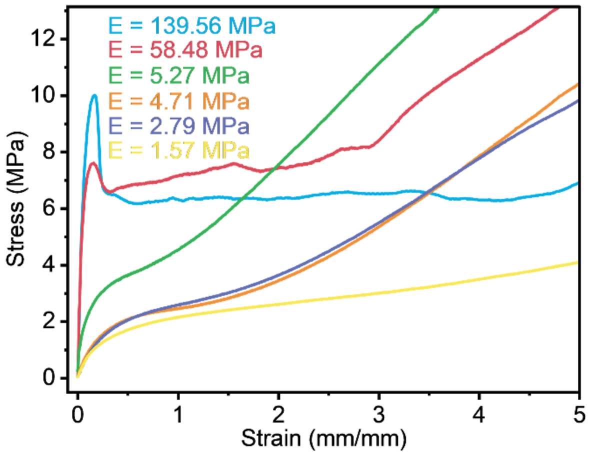

Fig. S11. The detailed magnification of the stress-strain curve for $PFPE_x$-PC_y.

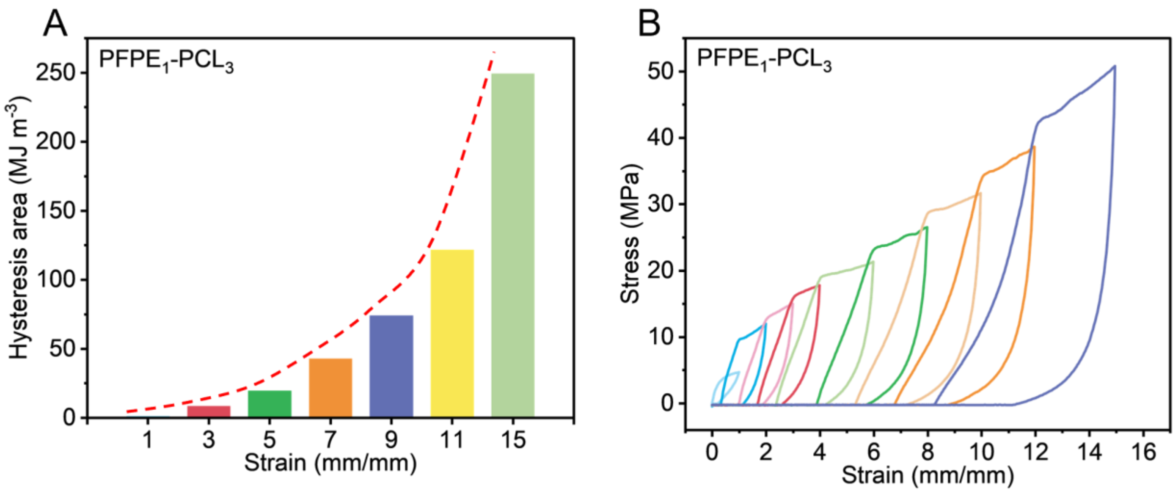

Fig. S12. Cyclic hysteresis loop. (A) Hysteresis area of $PFPE_1$-PCL_3 during cyclic stress-strain curves at varying strain levels with graded multi-sample. (B) Continuous cyclic stress-strain test with progressively increasing strains.

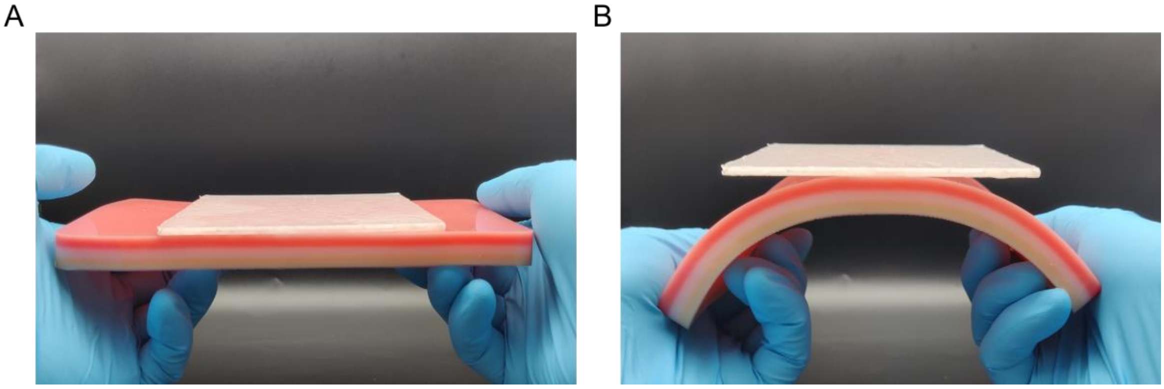

Fig. S13. Photograph comparing the elastic modulus of commercial SMP PCL with that of artificial skin tissue.

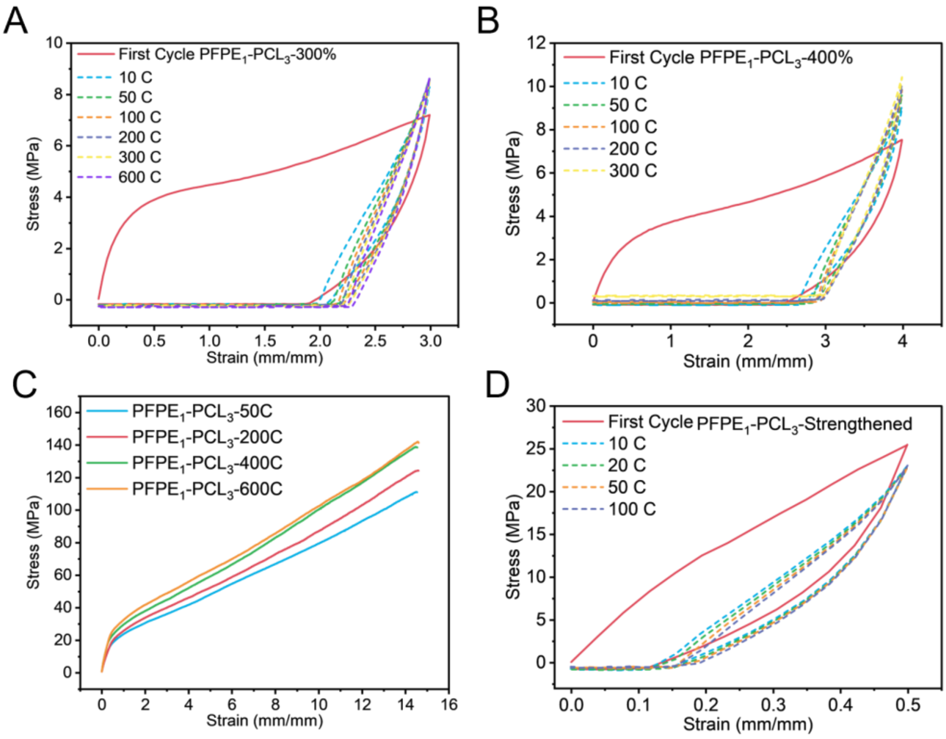

Fig. S14. Stress-strain curve. (A) Cyclic stress-strain curves of $PFPE_1$-PCL_3 under repetitive mechanical training at different strain showing self-strengthen properties by 600 cycles and 300 cycles (B). (C) Stress-strain curves of $PFPE_1$-PCL_3 after mechanical training at 300% strain with different cycles. (D) Cyclic stress-strain cures of $PFPE_1$-PCL_3 after mechanical training.

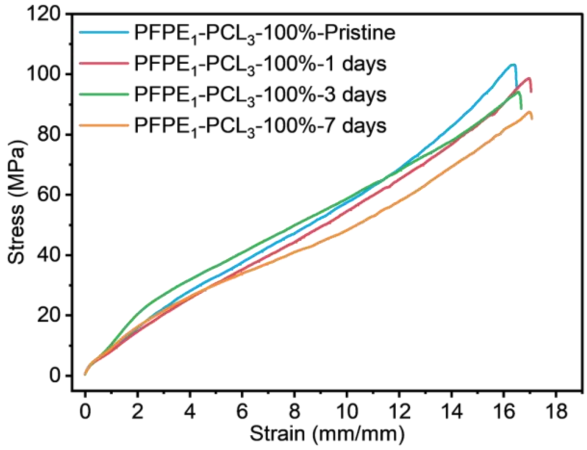

Fig. **S15.** The mechanical stability of PFPE$_1$-PCL$_3$ at room temperature after mechanical training at 100% strain.

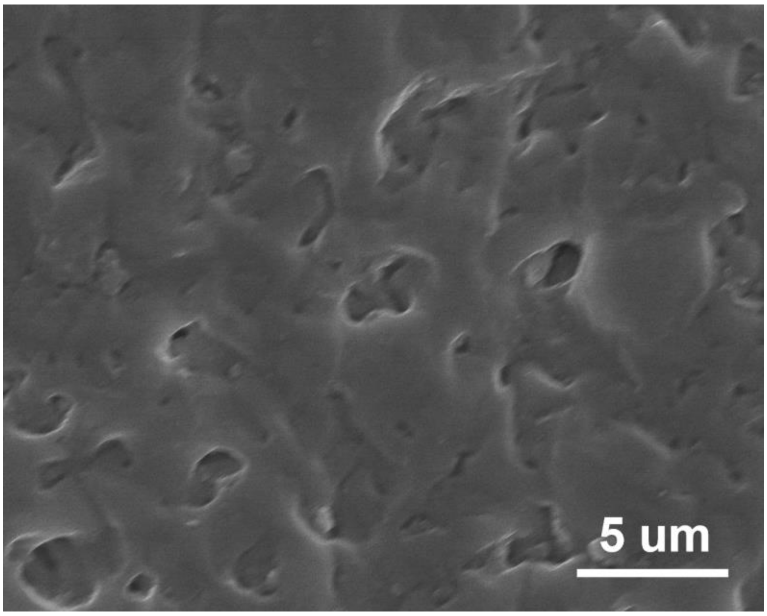

Fig. S16. SEM images of PFPE$_1$-PCL$_3$ before stretching.

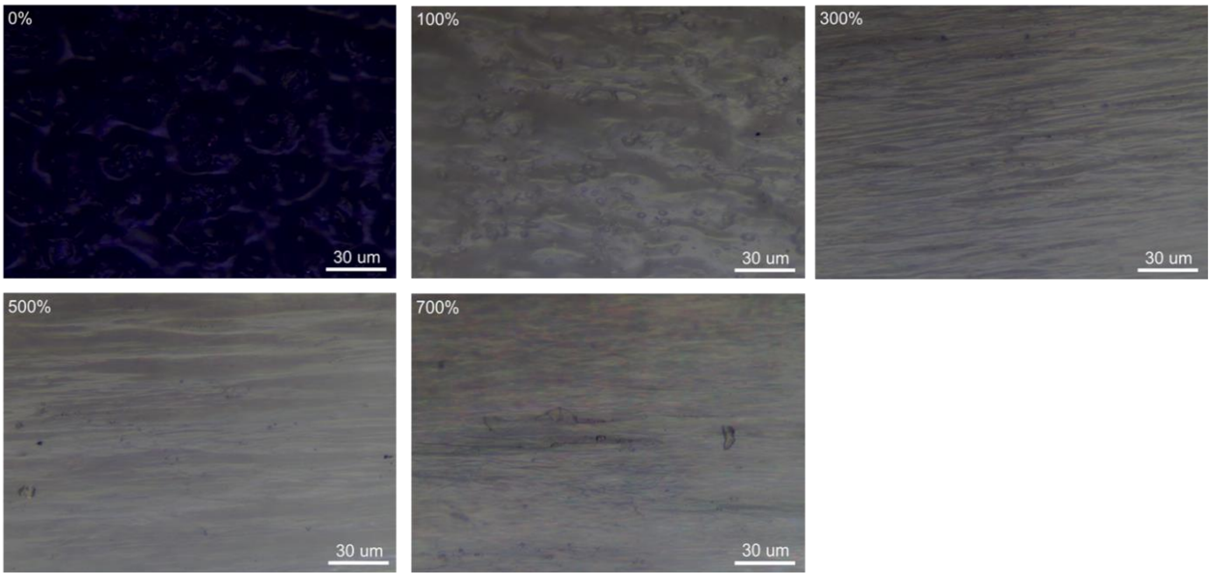

Fig. S17. POM images of PFPE$_1$-PCL$_3$ under different strain. The images show a transformation from an amorphous structure to a tightly aligned oriented fiber as the strain varied from 0% to 700%.

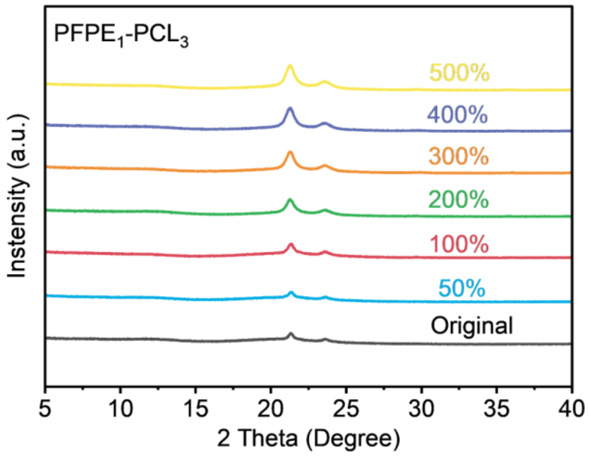

Fig. S18. XRD results of PFPE$_1$-PCL$_3$ after repetitive mechanical training at differ strain by 300 cycles.

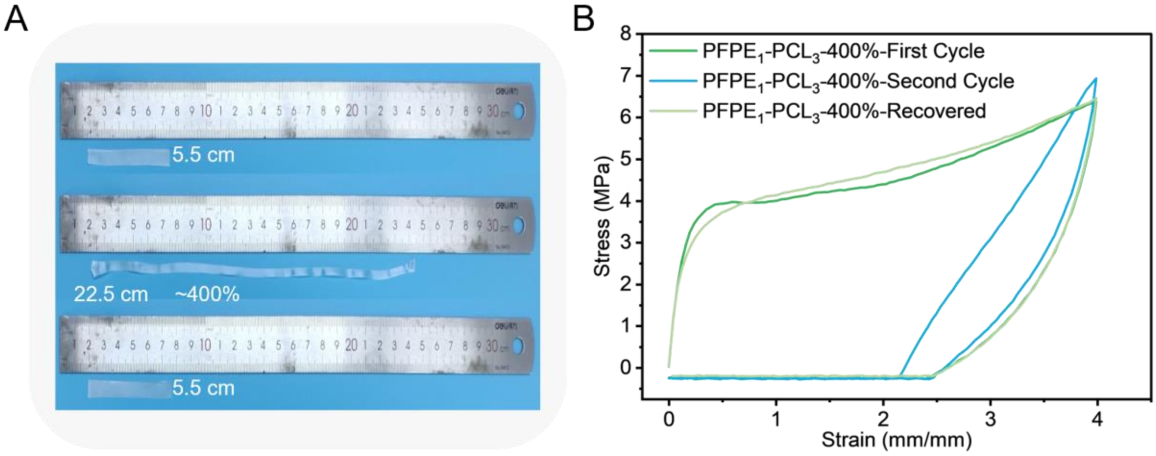

Fig. S19. Shape memory properties. (A) Picture of $PFPE_1$-PCL_3 at the original state, temporary state and shape-recovered state (after heating). (B) Cyclic tensile curves of $PFPE_1$-PCL_3 at first cycle, second cycle and recovered after heating.

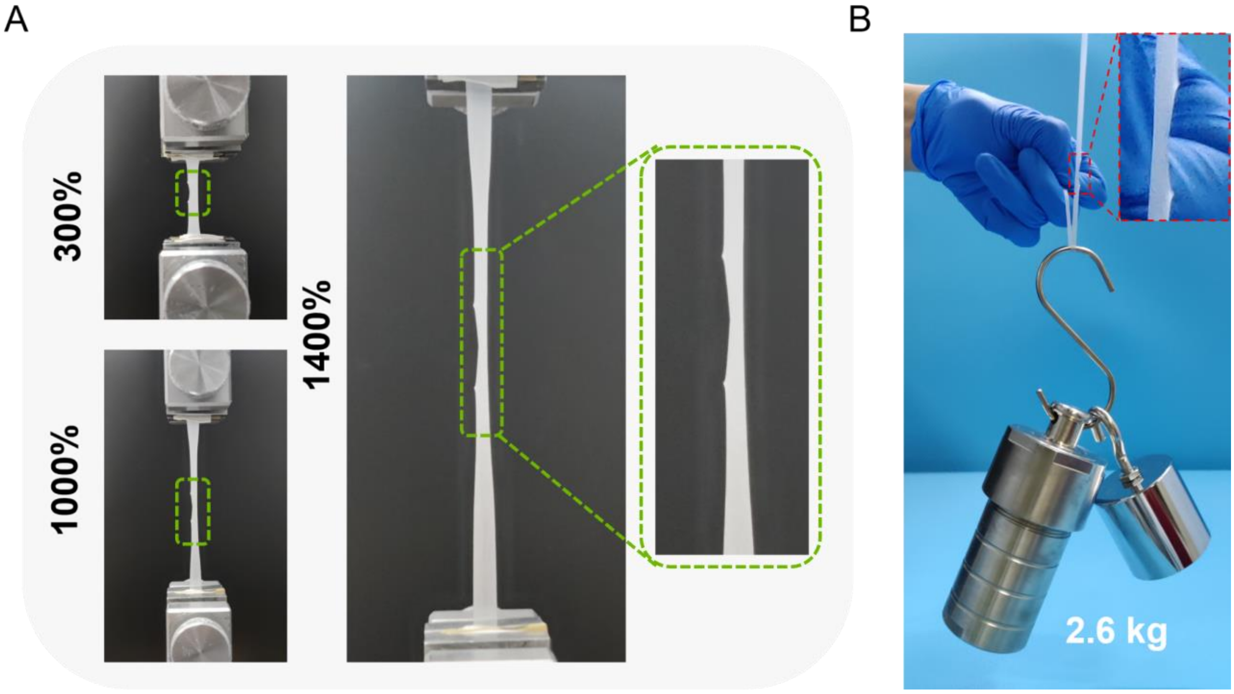

Fig. S20. Tear resistance test. (A) Pictures of the notched sample stretch to different strain. (B) Pictures of the notched $PFPE_1$-PCL_3 (0.2 g) can lift a weight of 2.6 kg.

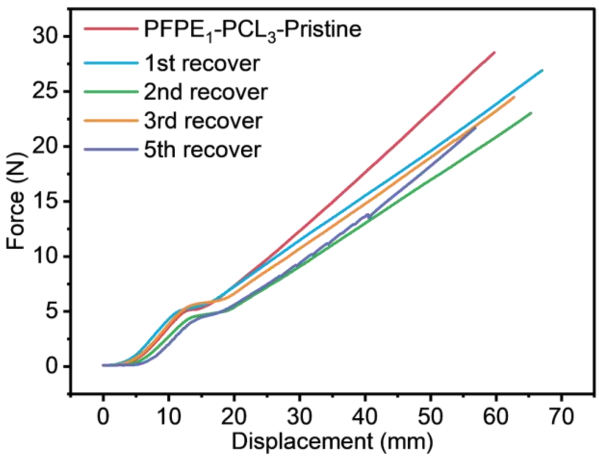

Fig. S21. Cyclic puncture tests of $PFPE_1$-PCL_3 before and after recovered by heating.

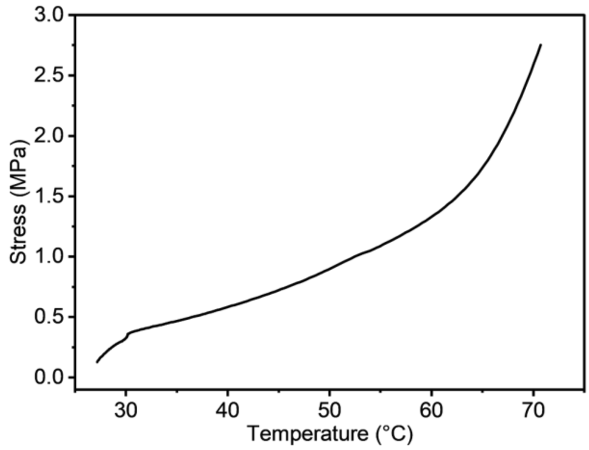

Fig. S22. Actuation force measured by DMA.

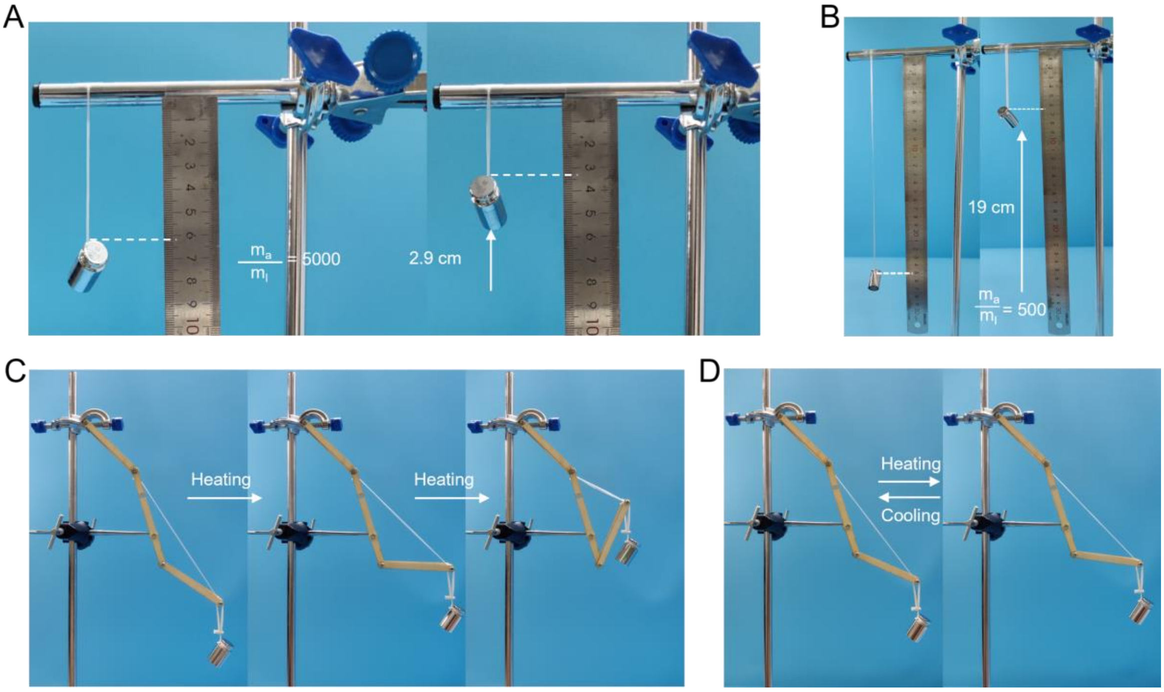

Fig. S23. Actuation performance of $PFPE_1$-PCL_3. (A) A pre-stretched film of $PFPE_1$-PCL_3 (4 mg) lift a weight (20g, 5000 times the elastomer) by 2.9 cm upon heating. (B) A pre-stretched $PFPE_1$-PCL_3 (40 mg) lifted a 20 g weight by 19 cm when heat applied. (C) A 20 mg elastomer can contract upon heating to lift a weight of 50 g rapidly with an actuation angle between the forearm and humerus of 102 °. (D) Reversible actuation of a pre-stretched $PFPE_1$-PCL_3 film to lift a weight of 50g under multiple heating-cooling process.

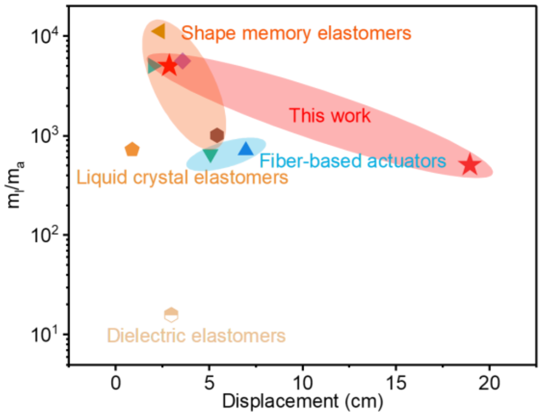

Fig. S24. Literature comparison of the actuation performance based on m_l/m_a and displacement among different kinds of actuators.

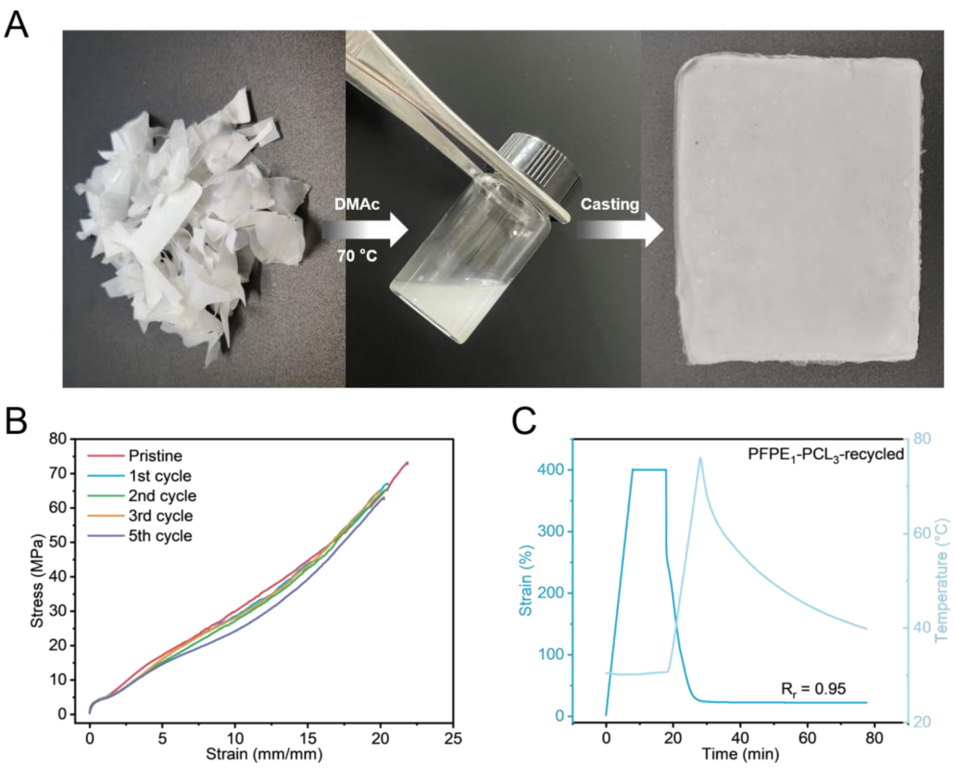

Fig. S25. Performance of PFPE$_1$-PCL$_3$ after recycle. (A) Images to illustrate the recycle process of the PFPE$_1$-PCL$_3$. (B) Stress-strain curves of PFPE$_1$-PCL$_3$ before and after recycle. (C) Shape memory performance tested by DMA after recycle. Fig. S25A illustrated the recycling and reprocessing process of PFPE$_1$-PCL$_3$. The samples were cut into pieces and then dissolved in the DMAc under 70 ℃. The obtained homogeneous mixtures were poured into a mold to obtain the recycled PFPE$_1$-PCL$_3$ elastomers after the solvent evaporation under room-temperature then for 70 ℃. After recycling and reprocessing for five times, the stress-strain curves of PFPE$_1$-PCL$_3$ are similar to the pristine one (Fig. S25B), except for a slight decrease in stress and strain at break, which probably owning to the formation of defect during the recycle process and the poorly rebuilding microstructures. The recycled sample also show shape memory behavior as shown in Fig. S25C.

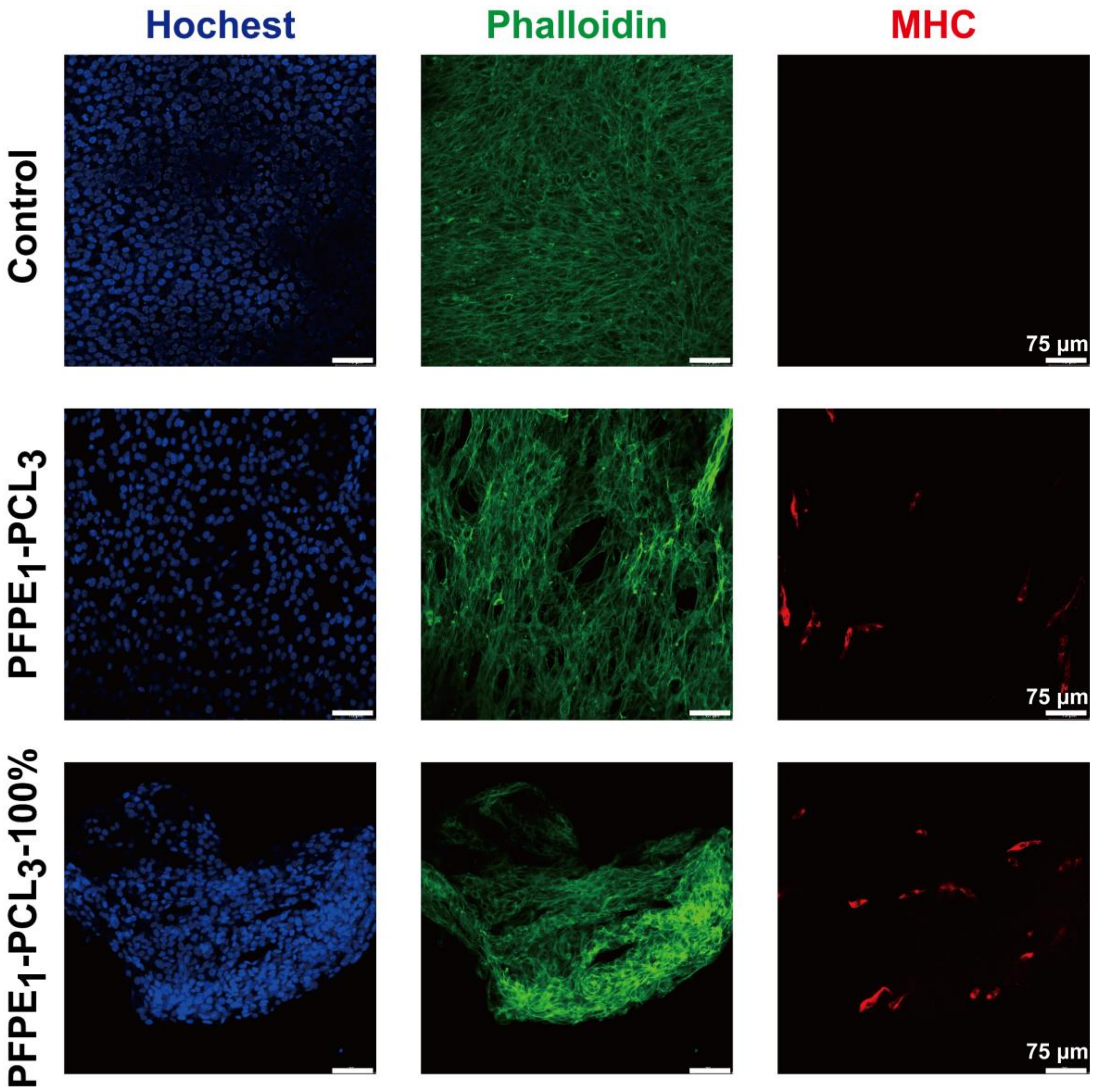

Fig. S26. MHC staining (red) and cytoskeleton staining by phalloidin (green) of C2C12 cells cultured on different sample for 7 days, (scale bar = 150 μm). Nuclei were stained as blue by Hoechst. All data are presented as mean ± SD. n = 4.

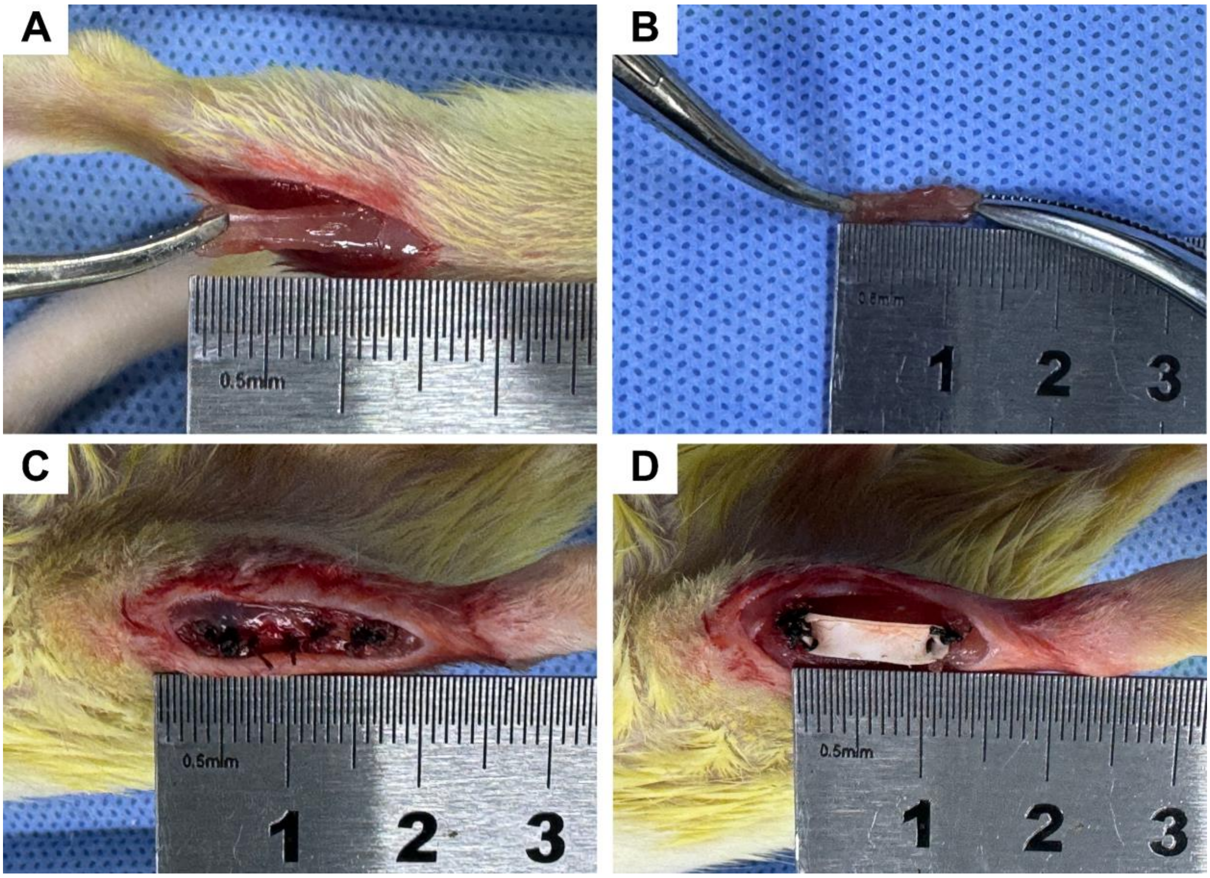

Fig. S27. The Surgical process of VML model.

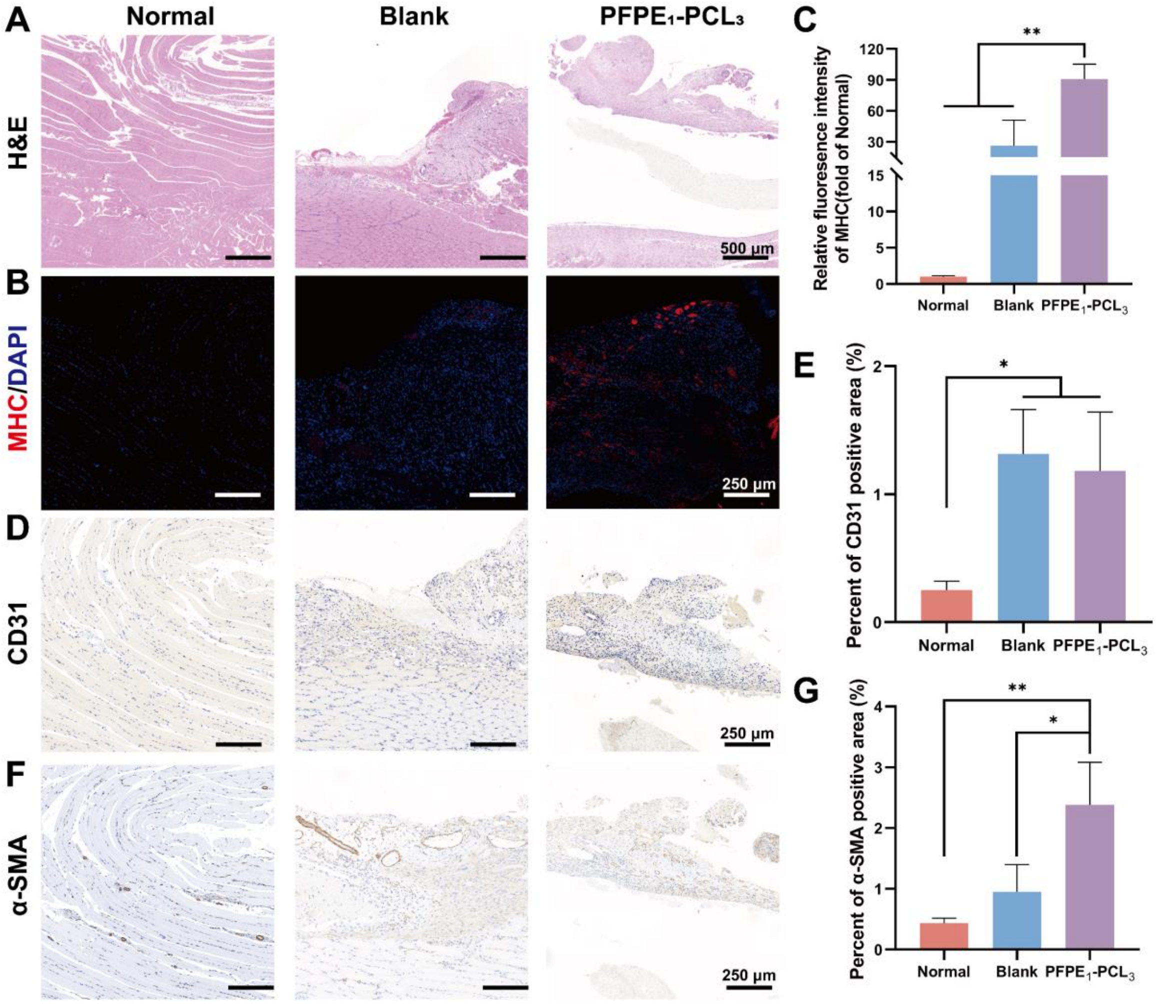

Fig. S28. Promotion of angiogenesis and muscle repair 1 weeks after $PFPE_1$-PCL_3 implantation in vivo. (A) Representative images of HE staining of TA muscle treated in different groups at week 1 postinjury (scale bar = 500 μm). (B) Representative images of immunofluorescence staining of TA remodeled muscle treated in different groups with MHC stained as red at week 1 postinjury (scale bar = 250 μm). (C) Quantitative analysis of immunofluorescence staining of MHC. (D) Representative images of CD31 staining of TA muscle treated in different groups at week 1 postinjury (scale bar = 250 μm). (E) Quantitative analysis of the expression of CD31. (F) Representative images of α-SMA staining of TA muscle treated in different groups at week 1 postinjury (scale bar = 250 μm). (G) Quantitative analysis of the expression of α-SMA. All data are presented as mean ± SD. ns $p > 0.05$, *$p < 0.05$, **$p < 0.01$. n = 4.

Table S1. Molecular weight of the $PFPE_x$-PCL_y.

Elastomer	M_n (g mol^{-1})	M_v (g mol^{-1})	M_w (g mol^{-1})	*PD*
$PFPE_0$-PCL_1	44156	83929	92359	2.09
$PFPE_1$-PCL_5	120135	270913	320881	2.67
$PFPE_1$-PCL_3	125113	352342	411668	2.57
$PFPE_1$-PCL_2	70428	211279	252194	2.13
$PFPE_1$-PCL_1	89721	226546	258481	2.18
$PFPE_2$-PCL_1	57937	98970	107265	1.85

Table S2. The T_g, T_m, melting enthalpy (ΔH_m), and crystallinity (X_c) of $PFPE_x$-PCL_y.

Materials	T_g (℃)	T_m (℃)	ΔH_m (J/g)	X_c (%)
$PFPE_0$-PCL_1	-55.1	55.5	68.23	50.54
$PFPE_1$-PCL_5	-55.9	47.8	53.42	39.57
$PFPE_1$-PCL_3	-55.0	42.2	27.41	20.30
$PFPE_1$-PCL_2	-55.1	40.7	24.48	18.13
$PFPE_1$-PCL_1	-54.9	36.2	12.12	8.97
$PFPE_2$-PCL_1	-54.8	22.0	7.87	5.82

※Crystallinity is estimated by comparing the melting enthalpy to that of 100% crystalline PCL (135 J/g) [7].

Table S3. Detailed mechanical performance of $PFPE_x$-PCL_y elastomers collected from tensile measurements.

Materials	Tensile stress [MPa]	Strain at break [%]	Elastic modulus [MPa]	Toughness [MJ m^{-3}]
$PFPE_0$-PCL_1	31.64 ± 1.09	2140 ± 112	139.56 ± 8.68	332.53 ± 38.81
$PFPE_1$-PCL_5	41.62 ± 4.89	2007 ± 101	58.48 ± 4.09	402.53 ± 32.93
$PFPE_1$-PCL_3	72.67 ± 3.19	2270 ± 88	5.27 ± 0.05	742.02 ± 23.98
$PFPE_1$-PCL_2	58.33 ± 1.71	2186 ± 46	4.71 ± 0.25	534.35 ± 24.91
$PFPE_1$-PCL_1	45.12 ± 2.38	2176 ± 86	2.79 ± 0.36	469.98 ± 29.67
$PFPE_2$-PCL_1	12.90 ±0.64	1909 ± 91	1.57 ± 0.13	138.59 ± 9.47

Table S4. Summary of the elastic modulus, mechanical strength, and toughness of this work, skeletal muscles, and shape memory polymers based on recent work.

**Materials**	**Elastic modulus (MPa)**	**Tensile strength (MPa)**	**Toughness (MJ m^{-3})**	**Ref.**
This work	5.27	72.67	742.02	This work
Skeletal muscle	0.1 - 0.3	1	-	[8, 9]
HEOMC-PU-3	247	12.3	55.5	[10]
CSSMP-5%	1147	84.1	312.7	[11]
DPUU-2000	33	84.18	322.84	[12]
PD_1	93.6	10.5	6.5	[13]
P-5-Zn	1129	55.6	226	[14]
PUU-0.5	76.37	95.56	308.63	[15]
PUU-a	132.7	9.04	17.3	[16]
PUN43	270.0	33.0	75.1	[17]
SMSP-0.1UPy	62	20.27	79.5	[18]
VESOV-0.7	317.4	18.7	10.8	[19]
4D-PTU	1200	61.39	2.7	[20]
$VHG_{7.5}$	116	41.3	205	[21]
BDO-BQDO-TPU-4	20.6	40.1	54.5	[22]
$A_2O_1P_6$	133	10.8	1.5	[23]
PU-DTDA20	131.4	13.4	33.4	[24]
S-Span-MDI-1	267	20.17	1.7	[25]
CA-PCL-DA-g-AH3	4.6	12.7	22.73	[26]
MPUF3	15.3	37.11	117.2	[27]
BE5	1.58	9.55	24.8	[28]

P(VI-co-MAAc) hydrogel	115	3.0	4.3	[29]
PAM/PF127 -30%	0.36	1.2	7.17	[30]
DCSM	336.2	14.2	0.83	[31]
LCE-COOH	272.6	11.68	0.52	[32]
DTPU-0.25-1k	1100	42.5	1.2	[33]
PEG-PCL/IL_1	13.7	2.94	5.5	[34]
5X	608.2	146.2	121.2	[35]
$H_6E_4P_6$	12.8	8.0	17.0	[36]
$PPeF_4$-AOH15-R1.8	332.1	55.1	206	[37]
PU&CNT	1160	49	139	[38]

Table S5 Work output and mechanical property comparison of different actuation materials.

**Materials**	**m_{load} /$m_{actuator}$**	**Elastic Modulus [MPa]**	**Displacement [cm]**	**Work Capacity [J/kg]**	**Reference**
$PFPE_1$-PCL_3	5000	5.27	2.9	1450	This work
	500	5.27	19	950	
Mammalian skeletal muscles	-	0.1 - 0.3	-	39	[39]
Fiber-based actuators	700	608.2	7	506	[35]
	650	10.64	5.1	40	[40]
Shape memory elastomers	5600	1200	3.6	2000	[41]
	11111	1147	2.4	2610	[11]
	5000	39.57	2	980	[42]
	1000	1160	5.45	823.91	[38]
Dielectric elastomers	20	1.3	3.0	15.6	[43]
Carbon nanotube yarns	-	294	-	2350	[44]
Liquid crystal elastomers	-	0.133	0.6	53	[45]
	714	1.02	0.9	63	[46]

Table S6. Summary of recovery durations for various scaffold materials in the treatment of volumetric muscle loss.

	**Materials**	**Recover time /(week)**	**Reference**
Synthetic polymers	$PFPE_1$-PCL_3	3	This work
Autografts	autografts	12	[47]
Synthetic polymers	OR-Exo	4	[48]
	PPBE-50	9	[49]
	NF5	4	[50]
	PCL/MLT	8	[51]
	PCM nanofibrous matrices	8	[52]
Natural polymer	Aligned Nanofibrous Collagen Scaffolds	8	[53]
	AC-DC	12	[54]
	AChR clusters	4	[55]
Extracellular matrix	adECM	4	[56]
	Bioconstructs	8	[57]
	ECM	24	[58]
	ECM fibers	8	[59]
	D-MA	8	[60]
	pFM	12	[61]
	ECM	4	[62]
	3D LNEM	10	[63]
	Aligned MEM	7	[64]
	PDSF	12	[65]
	EVs	4	[66]
Hydrogel	HA-CS hydrogels	4	[67]

	HA-g-PANI	4	[68]
	HM/GM/PFeC/Trp	4	[69]
	hydrogel-based matrix	4	[70]
	Pre-vascularized nerve hydrogel implants	8	[71]
Injectable hydrogel	mECM@IL4 + PM@IGF1	8	[72]
	IT-IC	4	[73]
	PAA-BLA	4	[74]
	FME	4	[75]
	MSNF/Gel	8	[76]
	C2C12-laden PLGA PMs	4	[77]
	micro-cryogel	5	[78]
	FPAu	4	[79]
3D printing hydrogel	FG IPN	24	[80]
	GelMA	8	[81]
	GelMA	5	[82]

Supplementary Movie

Movie S1

This video shows the puncture resistance test with a film of 0.4 mm.

Movie S2

This video shows that a film damaged by a needle can recover upon heating to 80 ℃.

Movie S3

This video shows that a pre-programmed film of 4 mg can lift a weight 5000 times its own weight by 2.9 cm upon heating in a second.

Movie S4

This video shows that a film of 40 mg can raise a weight 20 g by 19 cm.

Movie S5

This video shows that a pre-programmed film of 20 mg can contract to lift a weight of 50 g with an actuation angle between the forearm and humerus of 102 °.

Movie S6

This video shows the simple and efficiency program process of the artificial muscle.

Movie S7

This video shows that an arm lifting a 50 g weight displayed reversible action motions with an angle range higher than 15 °.

Movie S8

This video shows that $PFPE_1$-PCL_3 possesses mechanical properties comparable to muscle tissues and can facilitate early movement of limbs.

REFERENCES

1. Ducrot E, Chen Y, Bulters M *et al.* Toughening Elastomers with Sacrificial Bonds and Watching Them Break. *Science* 2014; **344**: 186-189.
2. Eugenis I, Wu D, Hu C *et al.* Scalable macroporous hydrogels enhance stem cell treatment of volumetric muscle loss. *Biomaterials* 2022; **290**: 121818.
3. Coleman MM, Zarian J. Fourier-transform infrared studies of polymer blends. II. Poly(ε-caprolactone)-poly(vinyl chloride) system. *J Polym Sci Polym Phys Ed* 1979; **17**: 837-850.
4. Kossack W, Seidlitz A, Thurn-Albrecht T *et al.* Molecular Order in Cold Drawn, Strain-Recrystallized Poly(ε-caprolactone). *Macromolecules* 2017; **50**: 1056-1065.
5. M. Shanthi P, J. Hanumantha P, Albuquerque T *et al.* Novel Composite Polymer Electrolytes of PVdF-HFP Derived by Electrospinning with Enhanced Li-Ion Conductivities for Rechargeable Lithium-Sulfur Batteries. *ACS Appl Energ Mater* 2018; **1**: 483-494.
6. Tan YJ, Godaba H, Chen G *et al.* A transparent, self-healing and high-κ dielectric for

low-field-emission stretchable optoelectronics. *Nat Mater* 2020; **19**: 182-188.

7. Nagata M, Yamamoto Y. Synthesis and characterization of photocrosslinked poly(ε-caprolactone)s showing shape-memory properties. *J Polym Sci A Polym Chem* 2009; **47**: 2422-2433.

8. Riccobelli D, Ambrosi D. Activation of a muscle as a mapping of stress-strain curves. *Extreme Mech Lett* 2019; **28**: 37-42.

9. Gillies AR, Lieber RL. Structure and function of the skeletal muscle extracellular matrix. *Muscle Nerve* 2011; **44**: 318-331.

10. Liu X, Wu J, Tang Z *et al.* Photoreversible bond-based shape memory polyurethanes with light-induced self-healing, recyclability, and 3d fluorescence encryption. *ACS Appl Mater Interfaces* 2022; **14**: 33829-33841.

11. Chen J, Wang Z, Yao B *et al.* Ultra-highly stiff and tough shape memory polyurea with unprecedented energy density by precise slight cross-linking. *Adv Mater* 2024;**36**: 2401178.

12. Wang X, Xu J, Zhang X *et al.* Molecularly engineered unparalleled strength and supertoughness of poly(urea-urethane) with shape memory and clusterization-triggered emission. *Adv Mater* 2022; **34**: 2205763.

13. Guo H, Puttreddy R, Salminen T *et al.* Halogen-bonded shape memory polymers. *Nat Commun* 2022; **13**: 7436.

14. Wang H-Q, Yu B-Y, Huang Z-Y *et al.* Shape-adaptive and recyclable radio-frequency devices based on polymer with variable stiffness. *Cell Re Phys Sci* 2024; **5**: 101882.

15. Wang X, Xu J, Zhang Y *et al.* A stretchable, mechanically robust polymer exhibiting shape-memory-assisted self-healing and clustering-triggered emission. *Nat Commun* 2023; **14**: 4712.

16. Wang S, Yang Y, Ying H *et al.* Recyclable, Self-Healable, and Highly Malleable Poly(urethane-urea)s with Improved Thermal and Mechanical Performances. *ACS Appl Mater Interfaces* 2020; **12**: 35403-35414.

17. Debnath S, Tiwary SK, Ojha U. Dynamic carboxylate linkage based reprocessable and self-healable segmented polyurethane vitrimers displaying creep resistance behavior and triple shape memory ability. *ACS Appl Polym Mater* 2021; **3**: 2166-2177.

18. Zhang S, Qin B, Xu J-F *et al.* Multi-recyclable shape memory supramolecular polyurea with long cycle life and superior stability. *ACS Mater Lett* 2021; **3**: 331-336.
19. Zhao X-L, Liu Y-Y, Weng Y *et al.* Sustainable epoxy vitrimers from epoxidized soybean oil and vanillin. *ACS Sustainable Chem Eng* 2020; **8**: 15020-15029.
20. Cui C, An L, Zhang Z *et al.* Reconfigurable 4D printing of reprocessable and mechanically strong polythiourethane covalent adaptable networks. *Adv Funct Mater* 2022; **32**: 2203720.
21. Zhao Z, Cao Z, Wu Z *et al.* Bicontinuous vitrimer heterogels with wide-span switchable stiffness-gated iontronic coordination. *Sci Adv* 2024; **10**: eadl2737.
22. Wang J, Lin X, Wang R *et al.* Self-healing, photothermal-responsive, and shape memory polyurethanes for enhanced mechanical properties of 3D/4D printed objects. *Adv Funct Mater* 2023; **33**: 2211579.
23. Yang X, Guo M, Yan J *et al.* A repeatable dual-encryption platform from recyclable thermosets with self-healing ability and shape memory effect. *Adv Funct Mater* 2022; **32**: 2205177.
24. Zhang C, Liang H, Liang D *et al.* Renewable castor-oil-based waterborne polyurethane networks: simultaneously showing high strength, self-healing, processability and tunable multishape memory. *Angew Chem Int Ed* 2021; **60**: 4289-4299.
25. Yan P, Zhao W, Zhang B *et al.* Inverse vulcanized polymers with shape memory, enhanced mechanical properties, and vitrimer behavior. *Angew Chem Int Ed* 2020; **59**: 13371-13378.
26. Zhao X, Dong R, Guo B *et al.* Dopamine-incorporated dual bioactive electroactive shape memory polyurethane elastomers with physiological shape recovery temperature, high stretchability, and enhanced C2C12 myogenic differentiation. *ACS Appl Mater Interfaces* 2017; **9**: 29595-29611.
27. Yang S, Wang S, Du X *et al.* Mechanically robust self-healing and recyclable flame-retarded polyurethane elastomer based on thermoreversible crosslinking network and multiple hydrogen bonds. *Chem Eng J* 2020; **391**: 123544.
28. Chen Y, Tang Z, Liu Y *et al.* Mechanically robust, self-healable, and reprocessable

elastomers enabled by dynamic dual cross-links. *Macromolecules* 2019; **52**: 3805-3812.
29. Zhu CN, Bai T, Wang H *et al.* Dual-encryption in a shape-memory hydrogel with tunable fluorescence and reconfigurable architecture. *Adv Mater* 2021; **33**: 2102023.
30. Li Y, Wang D, Wen J *et al.* Ultra-stretchable, variable modulus, shape memory multi-purpose low hysteresis hydrogel derived from solvent-induced dynamic micelle sea-island structure. *Adv Funct Mater* 2021; **31**: 2011259.
31. Zhou S-W, Zhou D, Gu R *et al.* Mechanically interlocked [c2]daisy chain backbone enabling advanced shape-memory polymeric materials. *Nat Commun* 2024; **15**: 1690.
32. Guo H, Ruoko T-P, Zeng H *et al.* Hydrogen-bonded liquid crystal elastomers combining shape memory programming and reversible actuation. *Adv Funct Mater* 2024; **34**: 2312068.
33. Liu B, Li H, Meng F *et al.* 4D printed hydrogel scaffold with swelling-stiffening properties and programmable deformation for minimally invasive implantation. *Nat Commun* 2024; **15**: 1587.
34. Wu S, Huang J, Jing S *et al.* Biodegradable shape-memory ionogels as green and adaptive wearable electronics toward physical rehabilitation. *Adv Funct Mater* 2023; **33**: 2303292.
35. Lang C, Lloyd EC, Matuszewski KE *et al.* Nanostructured block copolymer muscles. *Nat Nanotechnol* 2022; **17**: 752-758.
36. Wang Z, Heck M, Yang W *et al.* Tough peggels by in situ phase separation for 4D printing. *Adv Funct Mater* 2024; **34**: 2300947.
37. Sun D, Mo J, Liu W *et al.* Ultra-strong and tough bio-based polyester elastomer with excellent photothermal shape memory effect and degradation performance. *Adv Funct Mater*. **34**: 2403333.
38. Chen K, Li M, Yang Z *et al.* Ultra-large stress and strain polymer nanocomposite actuators incorporating a mutually-interpenetrated, collective-deformation carbon nanotube network. *Adv Mater* 2024; **36**: 2313354.
39. Mirvakili SM, Hunter IW. Artificial muscles: mechanisms, applications, and challenges. *Adv Mater* 2018; **30**: 1704407.
40. Kanik M, Orguc S, Varnavides G *et al.* Strain-programmable fiber-based artificial muscle.

Science 2019; **365**: 145-150.

41. Cooper CB, Nikzad S, Yan H *et al.* High energy density shape memory polymers using strain-induced supramolecular nanostructures. *ACS Central Sci* 2021; **7**: 1657-1667.

42. Xu Z, Liu Y-B, Wei D-W *et al.* Configurational entropy regulation in polyolefin elastomer/paraffin wax vitrimers by thermally responsive liquid-solid transition for force storage. *ACS Appl Mater Interfaces* 2023; **15**: 12423-12433.

43. Shi Y, Askounis E, Plamthottam R *et al.* A processable, high-performance dielectric elastomer and multilayering process. *Science* 2022; **377**: 228-232.

44. Mu J, Jung de Andrade M, Fang S *et al.* Sheath-run artificial muscles. *Science* 2019; **365**: 150-155.

45. Jiang Z, Abbasi BBA, Aloko S *et al.* Ultra-soft organogel artificial muscles exhibiting high power density, large stroke, fast response and long-term durability in air. *Adv Mater* 2023; **35**: 2210419.

46. Li S, Bai H, Liu Z *et al.* Digital light processing of liquid crystal elastomers for self-sensing artificial muscles. *Sci Adv* 2021; **7**: eabg3677.

47. Kim J, Kasukonis B, Roberts K *et al.* Graft alignment impacts the regenerative response of skeletal muscle after volumetric muscle loss in a rat model. *Acta Biomater* 2020; **105**: 191-202.

48. Jin S, Luo Z, Cai Y *et al.* Exosome-functionalized heterogeneous nanofibrous scaffolds repair bone defects accompanied by muscle injury. *Chem Eng J* 2024; **485**.

49. Fang Q, Wang D, Lin W *et al.* Highly Stretchable Piezoelectric Elastomer for Accelerated Repairing of Skeletal Muscles Loss. *Adv Funct Mater* 2024; **34**: 2313055.

50. Pham-Nguyen O-V, Son YJ, Kwon T-w *et al.* Preparation of stretchable nanofibrous sheets with sacrificial coaxial electrospinning for treatment of traumatic muscle injury. *Adv Healthc Mater* 2021; **10**: 2002228.

51. Xu Y, Chen X, Qian Y *et al.* Melatonin-based and biomimetic scaffold as muscle-ecm implant for guiding myogenic differentiation of volumetric muscle loss. *Adv Funct Mater* 2020; **30**: 2002378.

52. Kang MS, Yu Y, Park R *et al.* Highly aligned ternary nanofiber matrices loaded with

mxene expedite regeneration of volumetric muscle loss. *Nano-Micro Lett* 2024; **16**: 73.
53. Nakayama KH, Alcazar C, Yang G *et al.* Rehabilitative exercise and spatially patterned nanofibrillar scaffolds enhance vascularization and innervation following volumetric muscle loss. *npj Regen Med* 2018; **3**: 16.
54. Christensen KW, Turner J, Coughenour K *et al.* Assembled cell-decorated collagen (AC-DC) fiber bioprinted implants with musculoskeletal tissue properties promote functional recovery in volumetric muscle loss. *Adv Healthc Mater* 2022; **11**: 2101357.
55. Gilbert-Honick J, Iyer SR, Somers SM *et al.* Engineering 3D skeletal muscle primed for neuromuscular regeneration following volumetric muscle loss. *Biomaterials* 2020; **255**: 120154.
56. Liang W, Han M, Li G *et al.* Perfusable adipose decellularized extracellular matrix biological scaffold co-recellularized with adipose-derived stem cells and L6 promotes functional skeletal muscle regeneration following volumetric muscle loss. *Biomaterials* 2024; **307**: 122529.
57. Quarta M, Cromie M, Chacon R *et al.* Bioengineered constructs combined with exercise enhance stem cell-mediated treatment of volumetric muscle loss. *Nat Commun* 2017; **8**: 15613.
58. Sicari BM, Rubin JP, Dearth CL *et al.* An acellular biologic scaffold promotes skeletal muscle formation in mice and humans with volumetric muscle loss. *Sci Transl Med* 2014; **6**: 234ra258-234ra258.
59. Cell-Derived Extracellular Matrix fiber scaffolds improve recovery from volumetric muscle loss. *Tissue Engineering Part A* 2024; **30**: 181-191.
60. Wang S, Yan H, Fang B *et al.* A myogenic niche with a proper mechanical stress environment improves abdominal wall muscle repair by modulating immunity and preventing fibrosis. *Biomaterials* 2022; **285**: 121519.
61. Jin Y, Shahriari D, Jeon EJ *et al.* Functional skeletal muscle regeneration with thermally drawn porous fibers and reprogrammed muscle progenitors for volumetric muscle injury. *Adv Mater* 2021; **33**: 2007946.
62. Magarotto F, Sgrò A, Dorigo Hochuli AH *et al.* Muscle functional recovery is driven by

extracellular vesicles combined with muscle extracellular matrix in a volumetric muscle loss murine model. *Biomaterials* 2021; **269**: 120653.

63. Choi YS, Jeong E, Lee JS *et al.* Immunomodulatory scaffolds derived from lymph node extracellular matrices. *ACS Appl Mater Interfaces* 2021; **13**: 14037-14049.

64. Jin Y, Jeon EJ, Jeong S *et al.* Reconstruction of muscle fascicle-like tissues by anisotropic 3D patterning. *Adv Funct Mater* 2021; **31**: 2006227.

65. Zhang Q, Chiu Y, Chen Y *et al.* Harnessing the synergy of perfusable muscle flap matrix and adipose-derived stem cells for prevascularization and macrophage polarization to reconstruct volumetric muscle loss. *Bioact Mater* 2023; **22**: 588-614.

66. Magarotto F, Sgro A, Dorigo Hochuli AH *et al.* Muscle functional recovery is driven by extracellular vesicles combined with muscle extracellular matrix in a volumetric muscle loss murine model. *Biomaterials* 2021; **269**.

67. Narayanan N, Jia Z, Kim KH *et al.* Biomimetic glycosaminoglycan-based scaffolds improve skeletal muscle regeneration in a Murine volumetric muscle loss model. *Bioact Mater* 2021; **6**: 1201-1213.

68. Shi M, Dong R, Hu J *et al.* Conductive self-healing biodegradable hydrogel based on hyaluronic acid-grafted-polyaniline as cell recruitment niches and cell delivery carrier for myogenic differentiation and skeletal muscle regeneration. *Chem Eng J* 2023; **457**: 141110.

69. Shi M, Bai L, Xu M *et al.* Magnetically induced anisotropic conductive in situ hydrogel for skeletal muscle regeneration by promoting cell alignment and myogenic differentiation. *Chem Eng J* 2024; **484**: 149019.

70. Han WM, Anderson SE, Mohiuddin M *et al.* Synthetic matrix enhances transplanted satellite cell engraftment in dystrophic and aged skeletal muscle with comorbid trauma. *Sci Adv* 2018; **4**: eaar4008.

71. Wei S-Y, Chen P-Y, Tsai M-C *et al.* Enhancing the repair of substantial volumetric muscle loss by creating different levels of blood vessel networks using pre-vascularized nerve hydrogel implants. *Adv Healthc Mater* 2024; **13**: 2303320.

72. Li Y, Liu S, Zhang J *et al.* Elastic porous microspheres/extracellular matrix hydrogel injectable composites releasing dual bio-factors enable tissue regeneration. *Nat Commun*

2024; **15**: 1377.

73. Jin S, Choi H, Seong D *et al.* Injectable tissue prosthesis for instantaneous closed-loop rehabilitation. *Nature* 2023; **623**: 58-65.

74. Nam S, Lou J, Lee S *et al.* Dynamic injectable tissue adhesives with strong adhesion and rapid self-healing for regeneration of large muscle injury. *Biomaterials* 2024; **309**: 122597.

75. Zheng H, Cheng F, Guo D *et al.* Nanoenzyme-reinforced multifunctional scaffold based on $ti_3c_2t_x$ mxene nanosheets for promoting structure-functional skeletal muscle regeneration via electroactivity and microenvironment management. *Nano Lett* 2023; **23**: 7379-7388.

76. Wang L, Li T, Wang Z *et al.* Injectable remote magnetic nanofiber/hydrogel multiscale scaffold for functional anisotropic skeletal muscle regeneration. *Biomaterials* 2022; **285**: 121537.

77. Wang Y, Kankala RK, Cai Y-Y *et al.* Minimally invasive co-injection of modular micro-muscular and micro-vascular tissues improves in situ skeletal muscle regeneration. *Biomaterials* 2021; **277**: 121072.

78. Zhang Z, Zhao X, Wang C *et al.* Injectable conductive micro-cryogel as a muscle stem cell carrier improves myogenic proliferation, differentiation and in situ skeletal muscle regeneration. *Acta Biomater* 2022; **151**: 197-209.

79. Ge J, Li Y, Wang M *et al.* Engineering conductive antioxidative antibacterial nanocomposite hydrogel scaffolds with oriented channels promotes structure-functional skeletal muscle regeneration. *Chem Eng J* 2021; **425**: 130333.

80. Li T, Hou J, Wang L *et al.* Bioprinted anisotropic scaffolds with fast stress relaxation bioink for engineering 3D skeletal muscle and repairing volumetric muscle loss. *Acta Biomater* 2023; **156**: 21-36.

81. Eugenis I, Wu D, Rando TA. Cells, scaffolds, and bioactive factors: Engineering strategies for improving regeneration following volumetric muscle loss. *Biomaterials* 2021; **278**: 121173.

82. Hwangbo H, Lee H, Jin E-J *et al.* Photosynthetic cyanobacteria can clearly induce efficient muscle tissue regeneration of bioprinted cell-constructs. *Adv Funct Mater* 2023; **33**: 2209157.
